# Webcam-Based Real-Time Visual Feedback During Baduanjin Practice in Older Adults: 6-Week Pilot Randomized Study

**DOI:** 10.2196/101208

**Published:** 2026-09-14

**Authors:** Jeong-Woo Seo, Kahye Kim, Ji-Woo Seok, Minho Choi, Jin-Seung Choi

**Affiliations:** 1 Digital Health Research Division Korea Institute of Oriental Medicine Daejeon, Daejeon Republic of Korea; 2 Korean Convergence Medical Science University of Science and Technology Daejeon, Daejeon Republic of Korea; 3 School of Information and Communications Technology Convergence Engineering Department of Biomedical Engineering Konkuk University Chungju, Chungbuk Republic of Korea; 4 Research Institute of Biomedical Engineering Konkuk University Chungju, Chungbuk Republic of Korea

**Keywords:** Baduanjin qigong, visual feedback, computer vision, 2D pose estimation, older adults, pilot randomized study

## Abstract

**Background:**

Baduanjin qigong is a traditional mind-body exercise used to support balance and physical health in older adults. Age-related changes in proprioception may make accurate self-directed performance difficult without external guidance.

**Objective:**

The aim of this study is to explore whether webcam-based real-time visual feedback delivered during supervised laboratory sessions was associated with differences in webcam-derived 2D pose discrepancy and movement consistency during Baduanjin practice in older adults.

**Methods:**

A total of 31 older adults were enrolled, and 28 participants with complete analyzable records were included in this complete-case dataset (feedback group, n=14; nonfeedback group, n=14). All sessions were conducted face-to-face in a supervised motion-analysis laboratory. Weekly 2D pose-discrepancy values were analyzed using a linear mixed-effects model with fixed effects for group, categorical week, and the group-by-week interaction and a participant-specific random intercept. Joint- and movement-specific participant-level 6-week means were analyzed exploratorily using Welch independent-samples *t* tests. Holm correction was applied across 24 exploratory contrasts (6 week-specific, 8 joint-specific, and 10 movement-specific comparisons), and Hedges *g* and 95% CIs were reported. Participant-specific weekly slopes and within-participant variability were additionally examined to directly assess longitudinal error drift.

**Results:**

The linear mixed-effects model showed no significant group-by-week interaction (Wald *χ*^2^_5_=1.09; *P*=.96) and no significant overall week effect (Wald *χ*^2^_5_=6.40; *P*=.27). Averaged across 6 weeks, the feedback group had an estimated mean 2D pose discrepancy 1.20° lower than the nonfeedback group (95% CI −2.38° to −0.02°; *P*=.046), although this marginal pilot finding was sensitive to an analytic approach. No week-specific contrast remained significant after Holm adjustment. Nominal right elbow, right shoulder, and right knee differences did not survive global Holm correction. Form 3 showed a lower mean discrepancy in the feedback group (mean difference −3.70°, 95% CI −5.86° to −1.54°; Hedges g=−1.30; unadjusted *P*=.002; Holm-adjusted *P*=.04). Direct analyses of participant-specific slopes and within-participant SDs did not support a significant between-group difference in longitudinal error drift.

**Conclusions:**

In this small exploratory pilot study conducted under supervised laboratory conditions, the 6-week trajectories did not differ significantly between groups. A marginally lower average 2D pose discrepancy was observed in the feedback group across the 6 weeks, but no individual week- or joint-specific comparison remained significant after multiplicity adjustment. Form 3 was the only exploratory contrast that remained significant after global Holm correction. Direct longitudinal analyses did not demonstrate prevention of error drift. Larger studies using validated reference measurements, prespecified outcomes, and adequately powered longitudinal designs are required.

## Introduction

For decades, the rapid growth of the aging population has emerged as a global social issue, bringing significant challenges to public health and health care systems [1]. As aging progresses, the physiological and neurological functions of older adults gradually degenerate. According to Goble et al [2], the proprioceptive and somatosensory systems—which are crucial for detecting joint positions, muscle tension, and movements—become significantly blunted. This sensory degradation leads to a rapid decline in the asymmetric coordination abilities required to control multiple joints simultaneously, often resulting in impaired balance and an increased risk of falls [3]. Consequently, older adults experience severe limitations in self-perceiving the precise spatial positions of their extremities, especially when those limbs move outside their direct visual field. As pointed out by Yeh et al [4] and Mak et al [5], to compensate for this diminished somatosensory function, older adults develop a significantly heightened reliance on visual information for postural control and motor execution.

To mitigate such age-related physical and cognitive decline, Baduanjin qigong, a traditional mind-body exercise, has been widely studied for its potential benefits in older adults [6,7]. It has been associated with improvements in lower-limb strength, balance, and overall physical health through slow, continuous, and symmetrical or asymmetrical movements [6,7]. However, realizing these benefits requires precise kinematic execution [8]. Due to the aforementioned age-related proprioceptive decline, it is exceedingly difficult for older individuals to maintain accurate postures independently without the continuous, hands-on guidance of an expert. When older adults attempt complex multijoint movements without accurate internal sensory feedback, they instinctively resort to compensatory movement strategies, such as using excessive trunk flexion instead of proper arm extension [9]. As observed in motor control literature, the repeated use of these abnormal strategies limits true motor recovery [10]. A previous study has explicitly warned that self-training in the absence of appropriate extrinsic feedback induces “learned bad-use” [11]. This phenomenon involves maladaptive neuroplasticity, which instead solidifies incorrect compensatory movements, ingrains poor exercise habits, and ultimately prevents functional improvement [12].

Despite the importance of accurate movement execution, most Baduanjin studies have focused on changes in clinical indicators before and after supervised training rather than on quantitative assessment of the movement process itself [6]. Computer-vision and machine-learning approaches are increasingly used for posture-related assessment across heterogeneous sensor and video modalities, although data quality, model choice, task specificity, and population differences limit generalizability [13]. The Optimizing Performance through Intrinsic Motivation and Attention for Learning (OPTIMAL) theory of motor learning proposes that directing attention toward an external target or movement effect can support motor performance and learning [14,15]. In this study, the on-screen guide and color-coded cues were considered possible external visual anchors; however, attentional focus was not experimentally isolated as a mechanism.

This exploratory study evaluated a webcam-based real-time visual feedback system during Baduanjin practice in older adults. Unlike marker-based 3D motion-capture systems, the prototype used a standard red, green, and blue (RGB) webcam and a pose-tracking algorithm to calculate frontal-plane 2D projected joint-angle discrepancies relative to an expert guide video. The study was conducted face-to-face in a supervised laboratory and evaluated contact-free motion capture and feedback, not remote care. Potential home-based or telerehabilitation use is therefore a future application that requires separate feasibility, usability, safety, and effectiveness evaluation.

## Methods

### System Design and Implementation

#### System Overview and Architecture

The research prototype was an integrated motion-feedback platform that extracted participant landmarks using RGB camera-based pose tracking and compared projected joint angles frame-by-frame with an expert guide video. The workflow comprised data extraction, angle-discrepancy calculation, color-coded visual feedback, and data storage. The application was implemented in the Unity 3D environment and was operated by study staff during supervised laboratory sessions; participants did not use the system remotely or without supervision.

#### Intervention Access, Data Security, and Privacy

The prototype was operated on a local study workstation and was not publicly accessible. The recorded variables included webcam-derived landmark coordinates, timestamps, and calculated discrepancies.

#### Motion Capture and Joint Angle Analysis

During data extraction, participant movement was captured through a frontal RGB webcam, and MediaPipe Pose (Google) was used to estimate 33 body landmarks in the 2D image plane [16]. Video input was acquired at 30 frames per second, consistent with the Logitech C270 recording setting. Because only x- and y-coordinate projections from a single frontal camera were analyzed, the estimated landmarks and angles were sensitive to perspective, depth changes, self-occlusion, and out-of-plane rotation.

For each joint, a projected 2D angle was calculated from 3 landmark points. For points 1, 2, and 3, with point 2 representing the joint center and point 1 and point 3 representing the adjacent segment landmarks on either side of that joint in the MediaPipe skeletal model, the angle was calculated as θ = arccos[((point 1 − point 2)·(point 3 − point 2))/(||point 1 − point 2|| ||point 3 − point 2||)] × 180/π. The 8 analyzed joints were the left and right elbows, shoulders, knees, and hips. The participant-guide outcome was the absolute discrepancy between corresponding projected angles, not a validated anatomical joint-angle error. The system was not validated against 3D motion capture, electrogoniometry, or manual expert assessment in this study.

#### Temporal Alignment and Data Quality Processing

Participant and guide-video angles were compared on a frame-by-frame basis within the Unity session using the angle values available at each corresponding frame. Frames for which one or more landmarks required for the relevant 3-point angle were unavailable were not used for that joint-angle comparison. No additional time-warping procedure was applied; the comparison followed the timing of the participant's movement relative to the guide video during the session. Consequently, minor differences in movement speed were reflected in the frame-by-frame discrepancy values. No post hoc temporal realignment was introduced for the statistical analysis.

#### Camera and Recording Setup

All recordings were obtained in the Konkuk University motion-analysis laboratory using a Logitech C270 HD Webcam (Logitech International SA). The webcam supports a maximum resolution of 1280 × 720 pixels at 30 frames per second, uses a fixed-focus lens, and has a diagonal field of view of approximately 55°. The webcam was positioned in front of the participant so that the entire body was visible within the camera view (Figure 1).

Rather than applying a fixed camera height and participant-to-camera distance to all participants, the relative position between the participant and webcam was individually adjusted before measurement using an angle-based calibration procedure. Participants were asked to maintain predefined joint positions of approximately 90° of elbow flexion and 45° of knee flexion. These reference angles were first measured using a manual goniometer. The participant-to-camera distance and webcam position were then adjusted until the corresponding webcam-derived joint angles matched the goniometer-measured reference angles as closely as possible. Once an appropriate position was established, the relative positions of the participant and webcam were maintained throughout the recording session.

Participants were instructed to wear comfortable clothing that was not excessively loose in order to minimize obstruction of body contours and major joint landmarks. Recordings were performed under normal indoor laboratory lighting conditions with sufficient brightness for full-body landmark detection. A plain, unobstructed background was used to reduce visual interference with pose estimation. Before each session, study staff confirmed that the participant's entire body and major joint landmarks were clearly visible within the camera view and repeated the positioning check when necessary. Because the outcome was based on joint angles rather than linear distances, no body-size or skeletal-length normalization was applied. Once the goniometer-based positioning procedure was completed, the webcam remained fixed during the recording session. If camera position or full-body visibility was disturbed, recording was paused and the positioning check was repeated before continuing. Study staff operated the system and monitored participant safety throughout all sessions.

**Figure 1:**
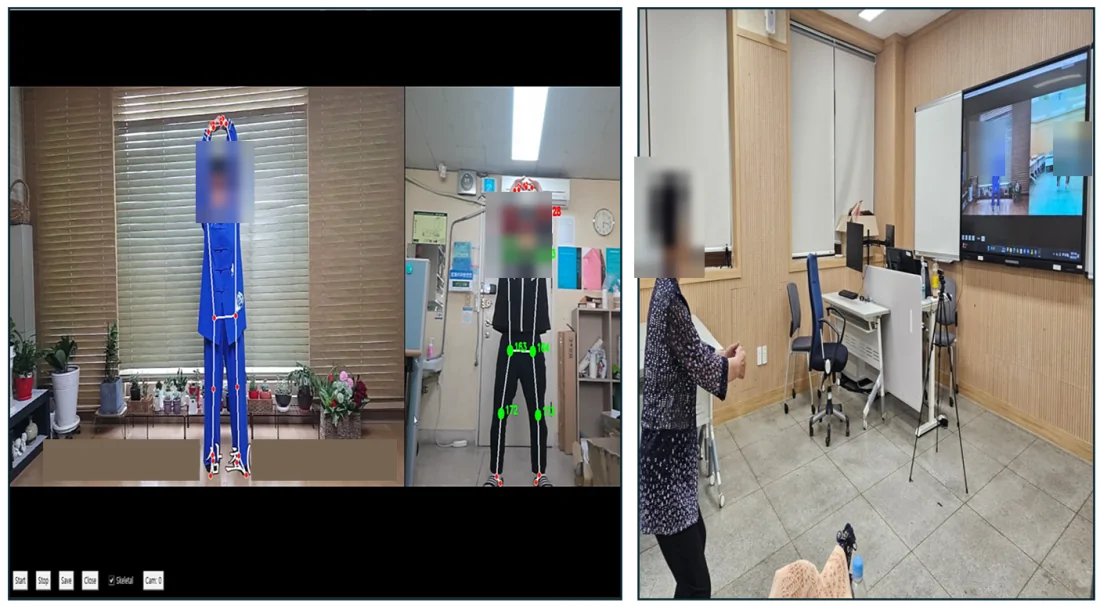
Deidentified screenshot of the supervised laboratory-based real-time motion feedback system.

#### Real-Time Feedback and Data Logging

During visualization, the Unity prototype used a programmed 10% angle-error tolerance setting for color-coded feedback. A joint was displayed in red when the participant-guide angle error exceeded this programmed tolerance and in green when it remained within the tolerance. The retained manuscript and analytic records identify the setting as a 10% angle-error threshold but do not preserve a more detailed normalization denominator or a separate clinical derivation for this parameter. Accordingly, the 10% value is reported as a prototype engineering tolerance setting rather than a clinically validated accuracy cutoff. After each session, landmark coordinates and time-series discrepancy values were stored for analysis. These kinematic values were not used as a substitute for attendance or program adherence.

#### Exercise Contents

The training content selected for this study is Baduanjin, a low-intensity aerobic qigong exercise ideal for older adults [7]. It consists of a 409-second sequence structured into 10 systematic phases, including the preparation phase (preliminary posture, 0-40 s); upper body and internal organ strengthening (pushing up the heavens, 41-94 s; drawing the bow, 95-134 s); digestive and core strengthening (separating heaven and earth, 135-158 s; looking backwards, 159-184 s; swaying the head and wagging the tail, 185-249 s); lower body and total vitality (touching the feet, 250-334 s; clenching the fists, 335-369 s; bouncing on the toes, 370-395 s); and finally, the closing phase (closing posture, 396-409 s) (Table 1).

**Table 1:** Key joint movements in the Baduanjin sequence.

Sequence	Movement name	Key anatomical joint movements
Preparation	Preliminary posture	Lower body: slight knee flexion; upper body: slight shoulder and elbow flexion
Form 1	Pushing up the heavens	Upward phase: shoulder flexion and elbow extension; downward phase: shoulder adduction and elbow flexion
Form 2	Drawing the bow	Lower body: knee flexion (horse-riding stance); upper body: horizontal abduction and elbow extension of the drawing arm
Form 3	Separating heaven and earth	Raised arm: shoulder flexion and elbow extension; lowered arm: shoulder extension
Form 4	Looking backwards	Lower body: knee flexion upon returning to the front; upper body: backward arm extension and cervical rotation
Form 5	Swaying the head	Spine: inducing slight hyperextension during 360° trunk and head rotation
Form 6	Touching the feet	Upper body: large shoulder and elbow rotation (from posterior to inferior)
Form 7	Clenching the fists	Upper body: arm extension forward combined with a 360° fist rotation
Form 8	Bouncing on the toes	Ankle and whole body: ankle plantarflexion (raising the heels) leading to whole-body extension
Closing	Closing posture	Whole body: releasing muscle tension and returning to a standing posture

#### Study Design, Setting, and Participants

A total of 31 older adults (20 women and 11 men) were enrolled. This complete-case dataset included 28 participants (feedback group, n=14; nonfeedback group, n=14). One participant withdrew, and 2 records were incomplete. Participants were assigned in a 1:1 ratio using a computer-generated random sequence. The feedback group viewed the same Baduanjin guide video as the nonfeedback group and additionally received real-time color-coded joint-discrepancy feedback. The nonfeedback group viewed the standard guide video without supplementary real-time feedback. Sessions were scheduled once weekly for 6 weeks, and the Baduanjin sequence lasted 409 seconds per performance. Participants and session staff could not be blinded because the feedback interface was visible.

Eligibility criteria were adults aged 60 years or older, ability to stand independently and perform low-intensity Baduanjin movements without physical assistance, ability to understand verbal or visual instructions, sufficient visual and auditory ability to perceive the guide video and feedback interface, no regular Baduanjin or similar structured mind-body training during the preceding 3 months, willingness to complete the 6-week program, and capacity to provide written informed consent. Exclusion criteria were severe musculoskeletal, neurological, cardiovascular, or respiratory conditions limiting safe participation; recent fracture, surgery, or acute pain affecting movement; balance impairment requiring physical assistance; cognitive or communication impairment preventing comprehension of procedures; severe visual impairment or color-vision deficiency preventing recognition of feedback cues; or participation in another exercise or rehabilitation intervention during the study period.

### Trial Registration

This study was not prospectively registered in a public clinical trial registry. At study initiation, it was conceived as an exploratory pilot investigation focused on the technical feasibility and preliminary kinematic evaluation of a webcam-based real-time visual feedback system rather than as a confirmatory efficacy trial; consequently, prospective trial registration was not undertaken before participant enrollment. The study nevertheless received institutional review board approval from Konkuk University (KKUIRB-202507-HR-092) before enrollment, and all participants provided written informed consent. We recognize that prospective registration would have enhanced transparency, and the absence of prospective registration is therefore explicitly acknowledged as a study limitation.

### Outcomes and Unit of Analysis

The measured outcome was the absolute discrepancy, in degrees, between participant and expert-guide joint angles projected in the frontal 2D image plane. It was a system-specific comparative measure and should not be interpreted as true anatomical joint-angle accuracy. Frame-level discrepancies were aggregated to participant-level session and weekly summaries.

### Pilot and Feasibility Outcomes

Feasibility-related domains considered for this pilot study included recruitment, retention of analyzable data, session completion, intervention exposure, usability, acceptability, technical interruptions, protocol deviations, and safety. Recruitment and analyzable-data retention could be summarized from the available study and analytic records. The analytic workbook used for the present reanalysis did not contain systematic participant-level measures of session completion, exposure, usability, acceptability, technical interruptions, protocol deviations, or adverse events; therefore, these domains were not analyzed quantitatively in this study.

### Sample Size

No formal power-based sample-size calculation was performed. The sample was determined by the number of eligible participants who could be recruited and complete the study during the available period. The study was therefore exploratory and was not powered to establish definitive clinical effectiveness.

### Statistical Analysis

Weekly mean 2D pose-discrepancy values were analyzed using a linear mixed-effects model with fixed effects for group (feedback vs nonfeedback), week (categorical and weeks 1-6), and the group-by-week interaction, with a participant-specific random intercept to account for within-participant correlation. Week 1 was the reference category, and the omnibus group-by-week interaction was the principal test of differential longitudinal change. Marginal between-group contrasts are reported as mean differences with 95% CIs. The estimated random-intercept intraclass correlation coefficient was also calculated. A Gaussian generalized estimating equation with exchangeable working correlation and robust covariance was fitted as a sensitivity analysis for the longitudinal outcome.

Week-specific model contrasts, joint-specific comparisons, and movement-specific comparisons were treated as exploratory. Joint- and movement-specific analyses were performed at the participant level using 6-week mean values. Welch independent-samples 2-tailed *t-*tests were used because group variances differed across several outcomes. To address multiplicity across the full set of exploratory comparisons, Holm correction was applied across 24 contrasts comprising 6 week-specific contrasts, 8 joint-specific contrasts, and 10 movement-specific contrasts. Standardized between-group effects for joint- and movement-specific comparisons are reported as Hedges g with 95% CIs for the mean differences. As supportive omnibus analyses of repeated outcomes within participants, separate random-intercept linear mixed-effects models were fitted to the joint and movement datasets. The joint model included fixed effects for group, joint (8-level categorical factor), and the group-by-joint interaction; the movement model included fixed effects for group, movement phase (10-level categorical factor), and the group-by-movement interaction. Participant was included as a random intercept, the models were estimated using restricted maximum likelihood, and the omnibus interaction terms were evaluated using Wald *χ*^2^ tests. These omnibus models were exploratory and were interpreted together with the multiplicity-adjusted individual contrasts. All tests were 2-sided.

To directly evaluate the proposed interpretation of longitudinal error drift, 3 participant-level analyses were conducted: (1) a linear slope across weeks 1-6 was estimated for each participant and compared between groups; (2) within-participant SD and range across the 6 weekly values were compared between groups; and (3) a random-intercept and random-slope mixed model with week treated as a continuous variable was fitted as a sensitivity analysis. These analyses distinguish true within-participant longitudinal change from between-participant dispersion observed at a single week.

The final analytic matrices contained no missing outcome values among the 28 analyzed participants (168 weekly, 224 joint, and 280 movement observations were complete). Therefore, missing-data imputation was not required within this complete-case dataset. However, the 3 excluded participants were not included in the uploaded analytic workbook; consequently, the missingness mechanism, Little’s test of missing completely at random (MCAR), multiple imputation, intention-to-treat analysis, and attrition sensitivity analyses for those participants could not be evaluated from the available data. Participant-level age was also unavailable, so a valid age-adjusted mixed model could not be fitted. Analyses were performed in Python 3.13.5 (Python Software Foundation) using SciPy 1.17.0 (SciPy Community) and statsmodels 0.14.6 (statsmodels Developers).

### Ethical Considerations

This was a single-center, 2-arm, parallel-group exploratory pilot randomized study with a 1:1 allocation ratio. The research protocol was approved by the institutional review board of Konkuk University (KKUIRB-202507-HR-092; July 25, 2025). All training and data collection were conducted face-to-face at the Konkuk University motion-analysis laboratory from July to September 2025, with study staff operating the system and monitoring participants. The study evaluated supervised, contact-free webcam measurement and visual feedback; it did not evaluate remote care or unsupervised home use. Written informed consent was obtained before participation.

## Results

### Participant Flow and Pilot Outcomes

Of the 31 enrolled participants, 28 were represented in this complete-case analytic dataset (feedback group, n=14; nonfeedback group, n=14), corresponding to 90.3% retention of analyzable records. One withdrawal and 2 incomplete records were documented. However, the timing of these 3 records relative to randomization and allocation and their group-specific assignment were not available in this analytic dataset. Accordingly, Figure 2 presents these records as a separate analytic-data note rather than assigning them to a pre- or postrandomization stage. Recruitment and analyzable-record retention are therefore reported descriptively; the other feasibility domains could not be quantified from the available analytic dataset. Baseline characteristics of the 28 participants included in the complete-case analysis are presented in Table 2.

Baseline characteristics are presented descriptively.

Significance tests were not performed.

**Figure 2:**
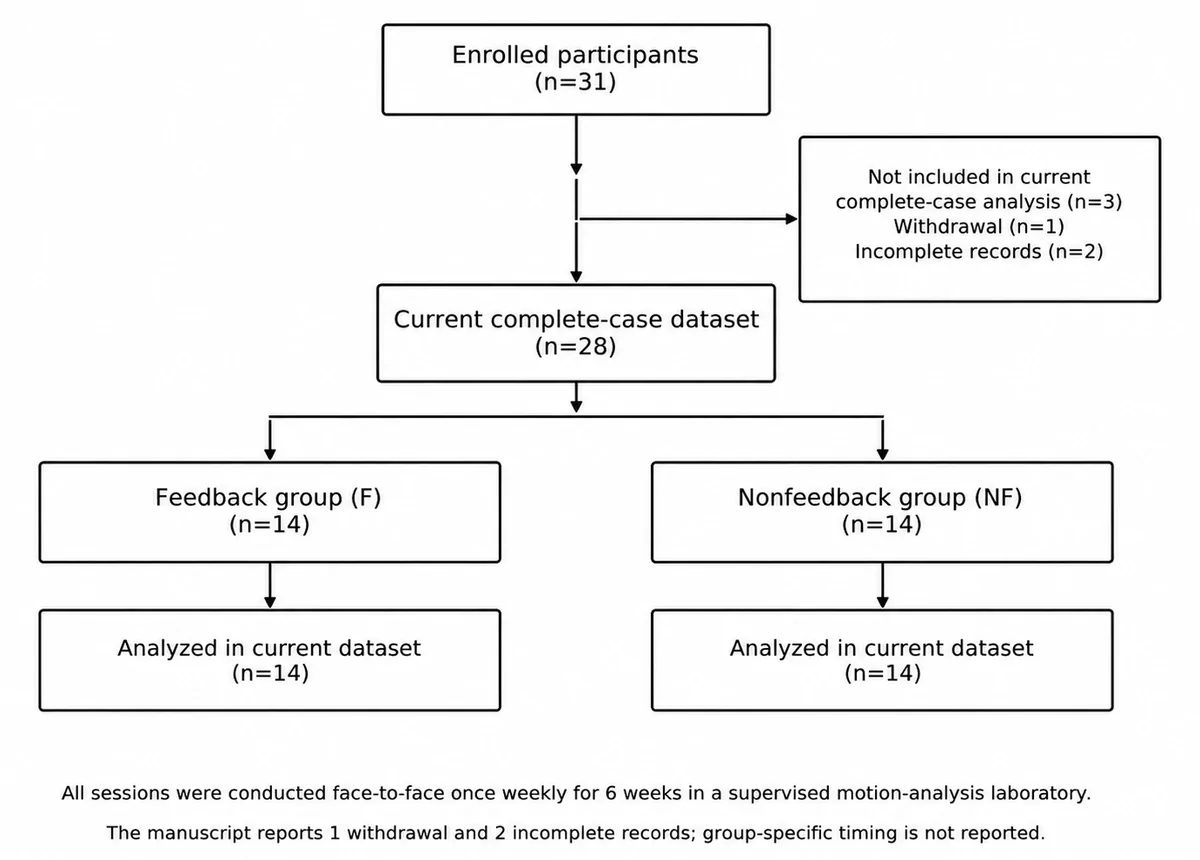
Participant flow diagram.

**Table 2:** Baseline characteristics of participants.a

Characteristic		Feedback group (n=14)	Nonfeedback group (n=14)
Sex, n			
	Male	5	5
	Female	9	9
Age (years), mean (SD)		80.60 (4.85)	75.92 (7.30)
Height (cm), mean (SD)		158.10 (7.20)	155.53 (7.34)
Weight (kg), mean (SD)		62.43 (14.14)	57.65 (7.07)

### Descriptive Weekly 2D Pose Discrepancy

Weekly participant-level mean 2D pose discrepancies and between-participant SDs are shown in Figure 3 and Table 3. Longitudinal inference was based on the participant-level linear mixed-effects model rather than separate independent tests at each week.

The feedback group showed numerically lower mean discrepancies than the nonfeedback group at all 6 weeks. The linear mixed-effects model showed no significant group-by-week interaction (Wald *χ*^2^_5_=1.09; *P*=.96), indicating no evidence that the 6-week trajectories differed between groups, and the overall categorical week effect was also not significant (Wald *χ*^2^_5_=6.40; *P*=.27). Averaged across the 6 weeks, the estimated marginal difference was −1.20° for feedback relative to nonfeedback (95% CI −2.38° to −0.02°; *P*=.046). The estimated random-intercept intraclass correlation coefficient (ICC) was 0.336. Because this was a small pilot sample, the marginal group effect was interpreted cautiously: a robust GEE sensitivity analysis gave the same point estimate (−1.20°, 95% CI −2.34° to −0.07°; *P*=.04), whereas a simple participant-level comparison of the 6-week means yielded a *P* value of .06. No week-specific contrast remained significant after Holm adjustment.

**Figure 3:**
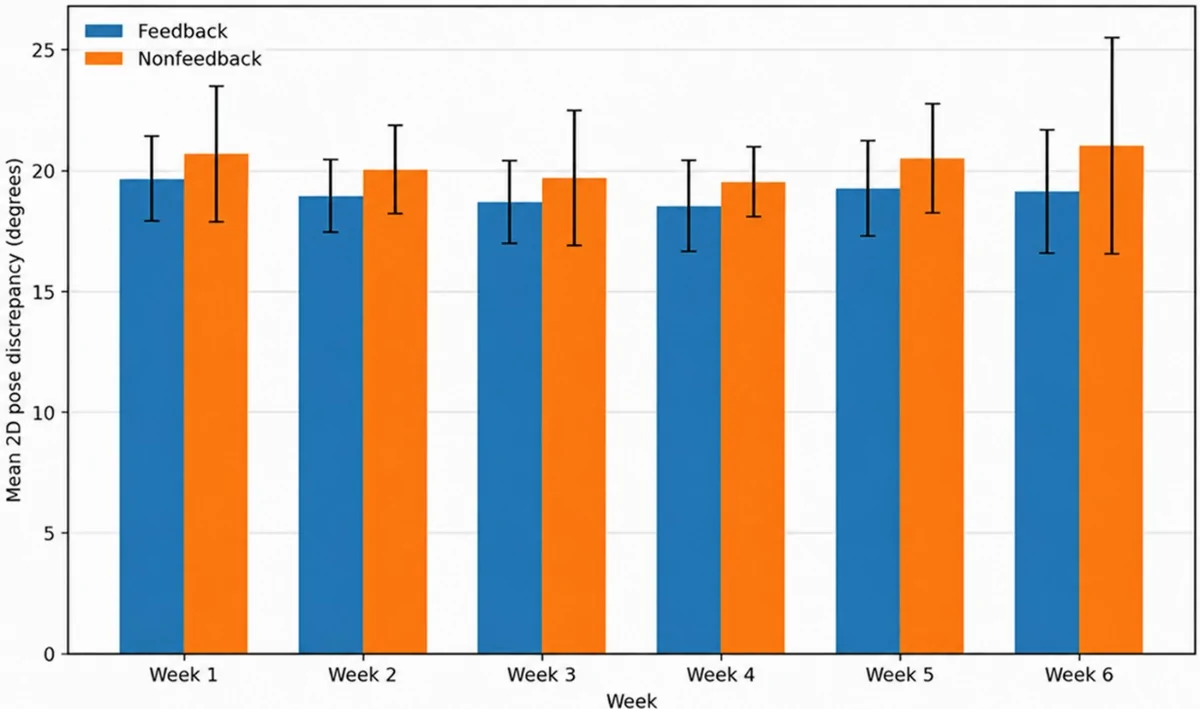
Descriptive weekly mean 2D pose discrepancy (weeks 1-6). Error bars represent between-participant SDs.

**Table 3:** Weekly mean 2D pose discrepancy and model-based between-group contrasts.^a^

Week	F^b^ group, mean (SD)	NF^c^ group, mean (SD)	F-NF difference (95% CI)	Raw *P* value	Holm-adjusted *P* value
Week 1	19.67 (1.76)	20.70 (2.81)	−1.04 (−2.80 to 0.73)	.25	>.99
Week 2	18.96 (1.50)	20.05 (1.82)	−1.09 (−2.86 to 0.67)	.23	>.99
Week 3	18.71 (1.71)	19.71 (2.79)	−1.00 (−2.77 to 0.77)	.27	>.99
Week 4	18.55 (1.88)	19.53 (1.44)	−0.98 (−2.75 to 0.78)	.28	>.99
Week 5	19.27 (1.96)	20.51 (2.25)	−1.23 (−3.00 to 0.53)	.17	>.99
Week 6	19.15 (2.55)	21.03 (4.47)	−1.88 (−3.65 to −0.11)	.04	.78

^a^Between-group differences and 95% CIs are model-based marginal contrasts from the linear mixed-effects model. Holm-adjusted *P* values control familywise error across the same global set of 24 exploratory contrasts (6 week-specific, 8 joint-specific, and 10 movement-specific comparisons).

^b^F: feedback.

^c^NF: nonfeedback.

### Longitudinal Error-Drift Sensitivity Analyses

Although the week-6 between-participant SD was larger in the nonfeedback group than in the feedback group (4.47° vs 2.55°), direct participant-level analyses did not demonstrate a significant difference in longitudinal drift. Mean participant-specific weekly slopes were −0.052° (SD 0.527) per week in the feedback group and 0.080° (SD 0.851) per week in the nonfeedback group (between-group difference −0.132°/week, 95% CI −0.688 to 0.423; *P*=.63). Mean within-participant SDs across the 6 weeks were 1.15° (SD 0.82) and 1.77° (SD 1.62), respectively (difference −0.62°, 95% CI −1.64° to 0.39°; *P*=.21). A random-intercept and random-slope model likewise showed a nonsignificant group-by-linear-week coefficient of −0.132°/week (95% CI −0.657° to 0.393°; *P*=.62). Thus, the week-6 SD difference is descriptive and does not provide statistical evidence that feedback prevented error drift.

### Exploratory Joint-Specific 2D Pose Discrepancy

Exploratory comparisons were performed for the 8 joint-specific participant-level mean 2D pose discrepancies averaged over weeks 1-6. Welch independent-samples *t-*tests were used, and Holm correction was applied within the prespecified global family of 24 exploratory contrasts (6 week-specific, 8 joint-specific, and 10 movement-specific comparisons).

The right elbow, right shoulder, and right knee showed nominal unadjusted differences favoring the feedback group (mean differences −2.39°, −1.91°, and −0.82°; unadjusted *P*=.03, .045, and .04, respectively). However, none remained significant after global Holm correction (adjusted *P*=.70, .91, and .78, respectively). The corresponding Hedges g values were −0.85, −0.78, and −0.83. A repeated-joint mixed model showed no significant group-by-joint interaction (*χ*^2^_7_=7.42; *P*=.39). These results therefore do not support confirmed joint-specific correction effects (Table 4).

**Table 4:** Exploratory joint-specific 2D pose discrepancy with effect sizes and global Holm-adjusted *P* values.^a^

Joint	F^b^, mean (SD)	NF^c^, mean (SD)	F-NF difference (95% CI)	Raw *P* value	Holm-adjusted *P* value	Hedges g
Left elbow	28.96 (1.63)	30.43 (3.89)	−1.47 (−3.84 to 0.91)	.21	>.99	−0.48
Right elbow	28.64 (2.24)	31.04 (3.17)	−2.39 (−4.54 to −0.25)	.03	.70	−0.85
Left shoulder	22.95 (1.87)	24.41 (3.01)	−1.45 (−3.42 to 0.51)	.14	>.99	−0.56
Right shoulder	23.73 (1.95)	25.64 (2.76)	−1.91 (−3.78 to −0.04)	.045	.91	−0.78
Left knee	9.74 (0.66)	10.26 (1.40)	−0.51 (−1.38 to 0.36)	.23	>.99	−0.46
Right knee	11.05 (0.57)	11.87 (1.22)	−0.82 (−1.58 to −0.06)	.04	.78	−0.83
Left hip	14.09 (1.04)	15.12 (2.05)	−1.03 (−2.32 to 0.25)	.11	>.99	−0.62
Right hip	13.42 (1.10)	14.25 (1.81)	−0.83 (−2.01 to 0.34)	.16	>.99	−0.54

^a^*P* values are unadjusted Welch independent-samples tests. Holm-adjusted *P* values control familywise error across all 24 exploratory contrasts. Hedges g is reported as the standardized between-group effect. No joint-specific comparison remained significant after adjustment.

^b^F: feedback.

^c^NF: nonfeedback.

### Exploratory Movement-Specific 2D Pose Discrepancy

Exploratory comparisons were performed across the 10 Baduanjin sequence phases using participant-level mean 2D pose discrepancies averaged over weeks 1-6. Welch independent-samples *t* tests were used, with Holm correction applied across the same global family of 24 exploratory contrasts (Figure 4 and Table 5).

Form 3 (separating heaven and earth) showed a lower mean 2D pose discrepancy in the feedback group than in the nonfeedback group (24.09°, SD 1.99° vs 27.79°, SD 3.34°). The mean difference was −3.70° (95% CI −5.86° to −1.54°; Hedges *g*=−1.30; unadjusted *P*=.002), and this comparison remained significant after global Holm correction (adjusted *P*=.04). No other movement-specific comparison remained significant after adjustment. A repeated-movement mixed model did not show a significant group-by-movement interaction (*χ*^2^_9_=9.42; *P*=.40); therefore, the form 3 finding should be considered an exploratory movement-specific signal rather than definitive evidence of movement-selective efficacy.

**Figure 4:**
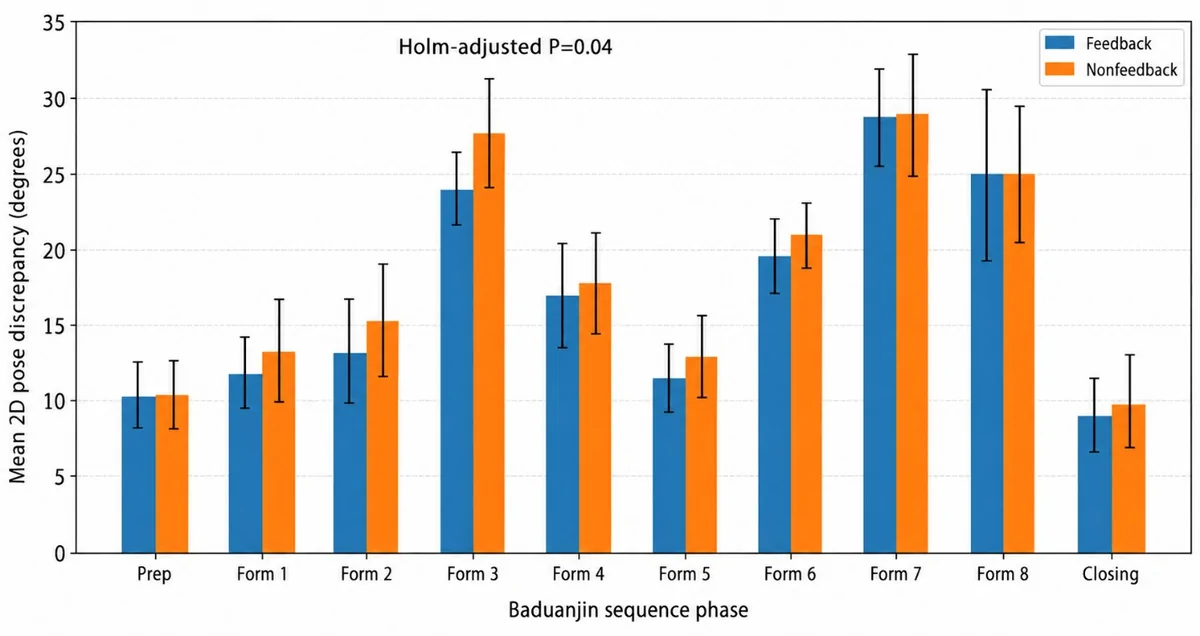
Exploratory movement-specific mean 2D pose discrepancy averaged over weeks 1-6.

**Table 5:** Exploratory movement-specific 2D pose discrepancy with effect sizes and global Holm-adjusted *P* values.^a^

Sequence	Movement	F^b,^ mean (SD)	NF^c^, mean (SD)	F-NF difference (95% CI)	Raw *P* value	Holm *P* value	Hedges *g*
Preparation	Preliminary posture	10.25 (1.77)	10.34 (1.67)	−0.09 (−1.43 to 1.24)	.89	>.99	−0.05
Form 1	Pushing up the heavens	11.53 (1.98)	12.86 (3.39)	−1.33 (−3.52 to 0.85)	.22	>.99	−0.47
Form 2	Drawing the bow	12.99 (3.32)	15.00 (3.41)	−2.05 (−4.66 to 0.57)	.12	>.99	−0.59
Form 3	Separating heaven and earth	24.09 (1.99)	27.79 (3.34)	−3.70 (−5.86 to −1.54)	.002	.04	−1.30
Form 4	Looking backwards	16.82 (3.29)	17.53 (3.45)	−0.71 (−3.33 to 1.91)	.58	>.99	−0.20
Form 5	Swaying the head	11.44 (1.38)	12.69 (2.28)	−1.26 (−2.74 to 0.22)	.09	>.99	−0.65
Form 6	Touching the feet	19.54 (2.00)	20.97 (2.07)	−1.42 (−3.01 to 0.16)	.08	>.99	−0.68
Form 7	Clenching the fists	28.87 (2.78)	29.20 (3.56)	−0.33 (−2.82 to 2.16)	.79	>.99	−0.10
Form 8	Bouncing on the toes	25.12 (6.20)	25.04 (4.60)	0.09 (−4.17 to 4.35)	.97	>.99	0.02
Closing	Closing posture	9.05 (1.52)	9.78 (2.80)	−0.73 (−2.50 to 1.05)	.40	>.99	−0.31

^a^*P* values are unadjusted Welch independent-samples tests. Holm-adjusted *P* values control familywise error across all 24 exploratory contrasts. Hedges *g* is reported as the standardized between-group effect. Form 3 was the only movement-specific comparison that remained significant after adjustment.

^b^F: feedback.

^c^NF: nonfeedback.

## Discussion

### Principal Findings

This exploratory pilot randomized study examined a webcam-based real-time visual feedback prototype during supervised laboratory-based Baduanjin practice in older adults. The outcome was webcam-derived 2D pose discrepancy relative to an expert guide video, not validated anatomical joint-angle accuracy. Computer-vision–based posture assessment has been increasingly investigated across different populations and application settings; however, measurement validity, task specificity, camera configuration, and population characteristics remain important methodological considerations [13,17]. The study did not evaluate remote delivery, home use, clinical effectiveness, or an isolated attentional-focus mechanism.

The participant-level longitudinal model did not identify a significant group-by-week interaction, indicating that the trajectories of weekly 2D pose discrepancy were not demonstrably different between groups over the 6 weeks. The estimated average difference across weeks favored the feedback group by 1.20°, but this was a marginal pilot result whose statistical significance varied slightly across sensitivity analyses. Accordingly, the effect should be interpreted in terms of magnitude and uncertainty rather than as definitive longitudinal efficacy. The larger week-6 SD in the nonfeedback group reflected between-participant heterogeneity at that time point. Direct analyses of participant-specific slopes, within-participant SDs, and a random-slope mixed model were nonsignificant; therefore, the data do not support a statistically demonstrable prevention of error drift.

The right elbow, right shoulder, and right knee comparisons were nominally significant before multiplicity adjustment but did not remain significant after global Holm correction across the 24 exploratory contrasts. The repeated-joint mixed model also showed no significant group-by-joint interaction. Accordingly, these joint-specific differences should not be interpreted as established correction effects or as evidence of lower-limb stability or fall-prevention benefit. They should instead be regarded as exploratory observations requiring replication using validated reference measurements and adequately powered samples. Previous work has emphasized the need to establish measurement validity when technology-based systems are used to quantify posture or movement [13,17].

Among the 10 movement phases, form 3 was the only exploratory contrast that remained significant after the conservative global Holm correction (adjusted *P*=.04; Hedges g=−1.30). However, the overall group-by-movement interaction was not significant, so this isolated contrast should be interpreted as a hypothesis-generating movement-specific signal rather than definitive evidence that feedback is selectively effective for asymmetric movements. Form 3 involves asymmetric upper-limb positioning, and external visual cues may influence motor performance by directing attention toward movement-related targets or effects, as described in motor-learning and attentional-focus literature [14,15,18]. Older adults may also rely more strongly on visual information as proprioceptive function declines [2,4,19]. A recent study in individuals with Down syndrome likewise demonstrated that attentional-focus conditions can influence gross motor performance, although differences in study population preclude direct generalization to older adults [20]. Therefore, the present design cannot determine whether the form 3 difference was caused by attentional focus, sensory reweighting, task complexity, or another mechanism.

Forms involving cervical or trunk rotation, depth changes, and self-occlusion remain especially sensitive to the limitations of a single frontal camera. Camera-based motion analysis can provide practical quantitative information, but its accuracy depends on camera geometry, landmark visibility, and the specific movement being assessed [17]. These limitations are particularly relevant for movements containing substantial out-of-plane rotation because 2D projected landmarks cannot fully represent 3D joint motion.

The study demonstrates the technical feasibility of collecting contact-free webcam-derived pose data and delivering real-time visual cues during supervised sessions. Technology-assisted exercise and feedback interventions have shown potential for supporting physical activity and exercise participation in older adults [21-24]. However, usability, adherence, safety, and effectiveness may differ substantially between supervised laboratory and unsupervised home environments [21,22,24]. This study therefore does not demonstrate unsupervised home feasibility, telerehabilitation effectiveness, or commercialization readiness.

Future studies should first validate the 2D discrepancy measure against 3D motion capture or another criterion method [17] and establish test-retest reliability under standardized camera conditions. Subsequent studies should evaluate usability, acceptability, technical reliability, adherence, and safety in real home settings, consistent with previous technology-assisted exercise research in older adults [21-24]. Larger randomized studies should also investigate whether external visual feedback influences motor performance through attentional-focus or sensory-reweighting mechanisms [14,15,18-20].

### Limitations

Several limitations should be considered. First, the sample was small and selected for a supervised laboratory study, limiting precision and generalizability; the marginal overall group difference across weeks was sensitive to analytic approach. Second, no confirmatory primary outcome, progression criterion, or formal power calculation is documented, and the trial was not prospectively registered. Third, the current analytic workbook contained complete data for 28 participants but did not include the 3 excluded participants; therefore, missingness mechanisms, multiple imputation, intention-to-treat analysis, and attrition sensitivity could not be evaluated. Fourth, participant-level age was unavailable for reanalysis despite a descriptive between-group age difference, precluding age-adjusted modeling. Fifth, a single frontal RGB camera provides only 2D projected landmarks, and criterion validity and test-retest reliability were not established. Sixth, 24 exploratory week-, joint-, and movement-specific contrasts required multiplicity control; no week-specific or joint-specific comparison remained significant after Holm adjustment, and only form 3 remained significant. Seventh, direct analyses of participant-specific slopes and within-participant variability did not support a statistical prevention-of-error-drift claim. Finally, attendance, intervention exposure, usability, acceptability, technical-failure metrics, and unsupervised home use remain insufficiently characterized from the available analytic data.

### Conclusions

In this small exploratory pilot study conducted under supervised laboratory conditions, the 6-week trajectories of webcam-derived 2D pose discrepancy did not differ significantly between feedback and nonfeedback groups. The feedback group showed a marginally lower average discrepancy across the 6 weeks, but no individual week or joint-specific comparison remained significant after multiplicity adjustment. Form 3 was the only exploratory contrast that remained significant after global Holm correction. Direct longitudinal analyses did not demonstrate that feedback prevented error drift.

The findings support further technical and methodological development rather than definitive claims of clinical effectiveness. Future studies should prespecify a primary outcome, recruit an adequately powered sample, retain all randomized participants for intention-to-treat analysis, collect participant-level covariates such as age and a true preintervention baseline, validate the webcam-derived measure against a reference method, and evaluate usability, technical reliability, and unsupervised home use before telerehabilitation or home-health applications are inferred.

## Appendix

Multimedia Appendix 1 CONSORT-eHEALTH checklist (V 1.6.1).

## Abbreviations

ICC: intraclass correlation coefficient
MCAR: missing completely at random
OPTIMAL: Optimizing Performance through Intrinsic Motivation and Attention for Learning
RGB: red, green, and blue

## Data Availability

All data are available upon reasonable request to the corresponding author (JWS; jwseo02@kiom.re.kr).
