# Supplementary material for: Webcam-Based Real-Time Visual Feedback During Baduanjin Practice in Older Adults: 6-Week Pilot Randomized Study
Source: JMIR Form Res. 2026 Sep 14;10:e101208. doi: 10.2196/101208 (PMC13620434; doi:10.2196/101208)
Supplement: Supplementary file 1 [file formative_v10i1e101208_app1.pdf]

CONSORT-EHEALTH (V 1.6.1) - Submission/Publication Form

The CONSORT-EHEALTH checklist is intended for authors of randomized trials evaluating web-based and Internet-based applications/interventions, including mobile interventions, electronic games (incl multiplayer games), social media, certain telehealth applications, and other interactive and/or networked electronic applications. Some of the items (e.g. all subitems under item 5 - description of the intervention) may also be applicable for other study designs.

The goal of the CONSORT EHEALTH checklist and guideline is to be
a) a guide for reporting for authors of RCTs,
b) to form a basis for appraisal of an ehealth trial (in terms of validity)

CONSORT-EHEALTH items/subitems are MANDATORY reporting items for studies published in the Journal of Medical Internet Research and other journals / scientific societies endorsing the checklist.

Items numbered 1., 2., 3., 4a., 4b etc are original CONSORT or CONSORT-NPT (non-pharmacologic treatment) items.
Items with Roman numerals (i., ii, iii, iv etc.) are CONSORT-EHEALTH extensions/clarifications.

As the CONSORT-EHEALTH checklist is still considered in a formative stage, we would ask that you also RATE ON A SCALE OF 1-5 how important/useful you feel each item is FOR THE PURPOSE OF THE CHECKLIST and reporting guideline (optional).

Mandatory reporting items are marked with a red *.
In the textboxes, either copy & paste the relevant sections from your manuscript into this form - please include any quotes from your manuscript in QUOTATION MARKS,
or answer directly by providing additional information not in the manuscript, or elaborating on why the item was not relevant for this study.

YOUR ANSWERS WILL BE PUBLISHED AS A SUPPLEMENTARY FILE TO YOUR PUBLICATION IN JMIR AND ARE CONSIDERED PART OF YOUR PUBLICATION (IF ACCEPTED).
Please fill in these questions diligently. Information will not be copyedited, so please use proper spelling and grammar, use correct capitalization, and avoid abbreviations.

DO NOT FORGET TO SAVE AS PDF _AND_ CLICK THE SUBMIT BUTTON SO YOUR ANSWERS ARE IN OUR DATABASE !!!

Citation Suggestion (if you append the pdf as Appendix we suggest to cite this paper in the caption):

응답을 수정 중입니다. 이 페이지의 URL을 공유하면 다른 사용자도 내 응답을 수정할 수 있습니다.
새 응답 작성하기

URL: http://www.jmir.org/2011/4/e126/
doi: 10.2196/jmir.1923
PMID: 22209829

jwseo0908@gmail.com 계정 전환

비공개

저장하려면 다시 제출하세요

* 표시는 필수 질문임

Your name *

First Last

JEONGWOO SEO

Primary Affiliation (short), City, Country *

University of Toronto, Toronto, Canada

Korea Institute of Oriental Medicine, Daejeon, S

Your e-mail address *

abc@gmail.com

jwseo02@kiom.re.kr

Title of your manuscript *

Provide the (draft) title of your manuscript.

Webcam-Based Real-Time Visual Feedback During Baduanjin Practice in Older Adults: A 6-Week Pilot Randomized Study

응답을 수정 중입니다. 이 페이지의 URL을 공유하면 다른 사용자도 내 응답을 수정할 수 있습니다.

새 응답 작성하기

Name of your App/Software/Intervention *

If there is a short and a long/alternate name, write the short name first and add the long name in brackets.

Webcam-Based Real-Time Visual Feedback Sy

Evaluated Version (if any)

e.g. "V1", "Release 2017-03-01", "Version 2.0.27913"

Research prototype; no formal version number

Language(s) *

What language is the intervention/app in? If multiple languages are available, separate by comma (e.g. "English, French")

English

URL of your Intervention Website or App

e.g. a direct link to the mobile app on app in appstore (itunes, Google Play), or URL of the website. If the intervention is a DVD or hardware, you can also link to an Amazon page.

내 답변

URL of an image/screenshot (optional)

내 답변

응답을 수정 중입니다. 이 페이지의 URL을 공유하면 다른 사용자도 내 응답을 수정할 수 있습니다.

새 응답 작성하기

Accessibility *

Can an enduser access the intervention presently?

- ◯ access is free and open
- ◯ access only for special usergroups, not open
- ◯ access is open to everyone, but requires payment/subscription/in-app purchases
- ◯ app/intervention no longer accessible
- ◉ 기타: Research prototype operated locally by study staff during supervised la

Primary Medical Indication/Disease/Condition *

e.g. "Stress", "Diabetes", or define the target group in brackets after the condition, e.g. "Autism (Parents of children with)", "Alzheimers (Informal Caregivers of)"

Older adults; age-related balance/postural and

Primary Outcomes measured in trial *

comma-separated list of primary outcomes reported in the trial

Webcam-derived 2D pose discrepancy relative

Secondary/other outcomes

Are there any other outcomes the intervention is expected to affect?

Exploratory joint-specific 2D pose discrepancy; movement-specific 2D pose discrepancy; participant-specific weekly slopes; within-participant variability/error-drift measures; analyzable-data retention. Other feasibility domains (session completion, exposure, usability, acceptability, technical interruptions, protocol deviations, and adverse events) were not available for quantitative analysis in the current analytic dataset.

응답을 수정 중입니다. 이 페이지의 URL을 공유하면 다른 사용자도 내 응답을 수정할 수 있습니다.

새 응답 작성하기

Recommended "Dose" *

What do the instructions for users say on how often the app should be used?

- ○ Approximately Daily
- ◉ Approximately Weekly
- ○ Approximately Monthly
- ○ Approximately Yearly
- ○ "as needed"
- ○ 기타:

Approx. Percentage of Users (starters) still using the app as recommended after 3 months *

- ◉ unknown / not evaluated
- ○ 0-10%
- ○ 11-20%
- ○ 21-30%
- ○ 31-40%
- ○ 41-50%
- ○ 51-60%
- ○ 61-70%
- ○ 71%-80%
- ○ 81-90%
- ○ 91-100%
- ○ 기타:

응답을 수정 중입니다. 이 페이지의 URL을 공유하면 다른 사용자도 내 응답을 수정할 수 있습니다.

새 응답 작성하기

Overall, was the app/intervention effective? *

- ( ) yes: all primary outcomes were significantly better in intervention group vs control
- ( ) partly: SOME primary outcomes were significantly better in intervention group vs control
- ( ) no statistically significant difference between control and intervention
- ( ) potentially harmful: control was significantly better than intervention in one or more outcomes
- (●) inconclusive: more research is needed
- ( ) 기타:

Article Preparation Status/Stage *

At which stage in your article preparation are you currently (at the time you fill in this form)

- ( ) not submitted yet - in early draft status
- ( ) not submitted yet - in late draft status, just before submission
- ( ) submitted to a journal but not reviewed yet
- (●) submitted to a journal and after receiving initial reviewer comments
- ( ) submitted to a journal and accepted, but not published yet
- ( ) published
- ( ) 기타:

응답을 수정 중입니다. 이 페이지의 URL을 공유하면 다른 사용자도 내 응답을 수정할 수 있습니다.

새 응답 작성하기

Journal *

If you already know where you will submit this paper (or if it is already submitted), please provide the journal name (if it is not JMIR, provide the journal name under "other")

- ◯ not submitted yet / unclear where I will submit this
- ◯ Journal of Medical Internet Research (JMIR)
- ◯ JMIR mHealth and UHealth
- ◯ JMIR Serious Games
- ◯ JMIR Mental Health
- ◯ JMIR Public Health
- ◉ JMIR Formative Research
- ◯ Other JMIR sister journal
- ◯ 기타:

Is this a full powered effectiveness trial or a pilot/feasibility trial? *

- ◉ Pilot/feasibility
- ◯ Fully powered

Manuscript tracking number *

If this is a JMIR submission, please provide the manuscript tracking number under "other" (The ms tracking number can be found in the submission acknowledgement email, or when you login as author in JMIR. If the paper is already published in JMIR, then the ms tracking number is the four-digit number at the end of the DOI, to be found at the bottom of each published article in JMIR)

- ◯ no ms number (yet) / not (yet) submitted to / published in JMIR
- ◉ 기타: 101208

응답을 수정 중입니다. 이 페이지의 URL을 공유하면 다른 사용자도 내 응답을 수정할 수 있습니다.

새 응답 작성하기

TITLE AND ABSTRACT

1a) TITLE: Identification as a randomized trial in the title

1a) Does your paper address CONSORT item 1a? *

I.e does the title contain the phrase "Randomized Controlled Trial"? (if not, explain the reason under "other")

- ○ yes
- ◉ 기타: The title identifies the study as randomized but intentionally uses the w

1a-i) Identify the mode of delivery in the title

Identify the mode of delivery. Preferably use “web-based” and/or “mobile” and/or “electronic game” in the title. Avoid ambiguous terms like “online”, “virtual”, “interactive”. Use “Internet-based” only if Intervention includes non-web-based Internet components (e.g. email), use “computer-based” or “electronic” only if offline products are used. Use “virtual” only in the context of “virtual reality” (3-D worlds). Use “online” only in the context of “online support groups”. Complement or substitute product names with broader terms for the class of products (such as “mobile” or “smart phone” instead of “iphone”), especially if the application runs on different platforms.

	1	2	3	4	5	
subitem not at all important	○	○	○	○	◉	essential

선택해제

응답을 수정 중입니다. 이 페이지의 URL을 공유하면 다른 사용자도 내 응답을 수정할 수 있습니다.

새 응답 작성하기

Does your paper address subitem 1a-i? *

Copy and paste relevant sections from manuscript title (include quotes in quotation marks "like this" to indicate direct quotes from your manuscript), or elaborate on this item by providing additional information not in the ms, or briefly explain why the item is not applicable/relevant for your study

"Webcam-Based Real-Time Visual Feedback During Baduanjin Practice in Older Adults: A 6-Week Pilot Randomized Study." The title explicitly identifies the webcam-based mode of delivery.

1a-ii) Non-web-based components or important co-interventions in title

Mention non-web-based components or important co-interventions in title, if any (e.g., “with telephone support”).

	1	2	3	4	5	
subitem not at all important	○	○	●	○	○	essential

선택해제

Does your paper address subitem 1a-ii?

Copy and paste relevant sections from manuscript title (include quotes in quotation marks "like this" to indicate direct quotes from your manuscript), or elaborate on this item by providing additional information not in the ms, or briefly explain why the item is not applicable/relevant for your study

The title identifies "Baduanjin Practice" as the exercise component. The supervised face-to-face laboratory setting is not stated in the title but is explicitly reported in the Abstract and Methods.

응답을 수정 중입니다. 이 페이지의 URL을 공유하면 다른 사용자도 내 응답을 수정할 수 있습니다.

새 응답 작성하기

1a-iii) Primary condition or target group in the title

Mention primary condition or target group in the title, if any (e.g., “for children with Type I Diabetes”) Example: A Web-based and Mobile Intervention with Telephone Support for Children with Type I Diabetes: Randomized Controlled Trial

	1	2	3	4	5	
subitem not at all important	○	○	○	○	◉	essential

선택해제

Does your paper address subitem 1a-iii? *

Copy and paste relevant sections from manuscript title (include quotes in quotation marks "like this" to indicate direct quotes from your manuscript), or elaborate on this item by providing additional information not in the ms, or briefly explain why the item is not applicable/relevant for your study

The target population is explicitly identified in the title as "Older Adults."

1b) ABSTRACT: Structured summary of trial design, methods, results, and conclusions

NPT extension: Description of experimental treatment, comparator, care providers, centers, and blinding status.

1b-i) Key features/functionalities/components of the intervention and comparator in the METHODS section of the ABSTRACT

Mention key features/functionalities/components of the intervention and comparator in the abstract. If possible, also mention theories and principles used for designing the site. Keep in mind the needs of systematic reviewers and indexers by including important synonyms. (Note: Only report in the abstract what the main paper is reporting. If this information is missing from the main body of text, consider adding it)

	1	2	3	4	5	
subitem not at all important	○	○	○	○	◉	essential

응답을 수정 중입니다. 이 페이지의 URL을 공유하면 다른 사용자도 내 응답을 수정할 수 있습니다.

새 응답 작성하기

Does your paper address subitem 1b-i? *

Copy and paste relevant sections from the manuscript abstract (include quotes in quotation marks "like this" to indicate direct quotes from your manuscript), or elaborate on this item by providing additional information not in the ms, or briefly explain why the item is not applicable/relevant for your study

"To explore whether webcam-based real-time visual feedback delivered during supervised laboratory sessions was associated with differences in webcam-derived 2D pose discrepancy and movement consistency during Baduanjin practice in older adults." The abstract also identifies the feedback and nonfeedback groups and the supervised laboratory setting. Detailed comparator content is provided in the Methods.

1b-ii) Level of human involvement in the METHODS section of the ABSTRACT

Clarify the level of human involvement in the abstract, e.g., use phrases like "fully automated" vs. "therapist/nurse/care provider/physician-assisted" (mention number and expertise of providers involved, if any). (Note: Only report in the abstract what the main paper is reporting. If this information is missing from the main body of text, consider adding it)

	1	2	3	4	5	
subitem not at all important	○	○	○	○	◉	essential

선택해제

Does your paper address subitem 1b-ii?

Copy and paste relevant sections from the manuscript abstract (include quotes in quotation marks "like this" to indicate direct quotes from your manuscript), or elaborate on this item by providing additional information not in the ms, or briefly explain why the item is not applicable/relevant for your study

"All sessions were conducted face-to-face in a supervised motion-analysis laboratory." The main Methods further state that study staff operated the system and monitored participants.

응답을 수정 중입니다. 이 페이지의 URL을 공유하면 다른 사용자도 내 응답을 수정할 수 있습니다.

새 응답 작성하기

1b-iii) Open vs. closed, web-based (self-assessment) vs. face-to-face assessments in the METHODS section of the ABSTRACT

Mention how participants were recruited (online vs. offline), e.g., from an open access website or from a clinic or a closed online user group (closed usergroup trial), and clarify if this was a purely web-based trial, or there were face-to-face components (as part of the intervention or for assessment). Clearly say if outcomes were self-assessed through questionnaires (as common in web-based trials). Note: In traditional offline trials, an open trial (open-label trial) is a type of clinical trial in which both the researchers and participants know which treatment is being administered. To avoid confusion, use “blinded” or “unblinded” to indicated the level of blinding instead of “open”, as “open” in web-based trials usually refers to “open access” (i.e. participants can self-enrol). (Note: Only report in the abstract what the main paper is reporting. If this information is missing from the main body of text, consider adding it)

	1	2	3	4	5	
subitem not at all important	○	○	○	○	◉	essential

선택해제

Does your paper address subitem 1b-iii?

Copy and paste relevant sections from the manuscript abstract (include quotes in quotation marks "like this" to indicate direct quotes from your manuscript), or elaborate on this item by providing additional information not in the ms, or briefly explain why the item is not applicable/relevant for your study

"All sessions were conducted face-to-face in a supervised motion-analysis laboratory." This was not a purely web-based or self-enrolled online trial. The manuscript does not specify the recruitment channel in sufficient detail to classify recruitment as online or offline.

응답을 수정 중입니다. 이 페이지의 URL을 공유하면 다른 사용자도 내 응답을 수정할 수 있습니다.

새 응답 작성하기

1b-iv) RESULTS section in abstract must contain use data

Report number of participants enrolled/assessed in each group, the use/uptake of the intervention (e.g., attrition/adherence metrics, use over time, number of logins etc.), in addition to primary/secondary outcomes. (Note: Only report in the abstract what the main paper is reporting. If this information is missing from the main body of text, consider adding it)

	1	2	3	4	5	
subitem not at all important	○	○	○	○	◉	essential

선택해제

Does your paper address subitem 1b-iv?

Copy and paste relevant sections from the manuscript abstract (include quotes in quotation marks "like this" to indicate direct quotes from your manuscript), or elaborate on this item by providing additional information not in the ms, or briefly explain why the item is not applicable/relevant for your study

The abstract reports: "Thirty-one older adults were enrolled, and 28 participants with complete analyzable records were included in the current complete-case dataset (F group, n=14; NF group, n=14)." System-use/adherence metrics were not systematically available in the analytic dataset and therefore were not reported as process outcomes.

1b-v) CONCLUSIONS/DISCUSSION in abstract for negative trials

Conclusions/Discussions in abstract for negative trials: Discuss the primary outcome - if the trial is negative (primary outcome not changed), and the intervention was not used, discuss whether negative results are attributable to lack of uptake and discuss reasons. (Note: Only report in the abstract what the main paper is reporting. If this information is missing from the main body of text, consider adding it)

	1	2	3	4	5	
subitem not at all important	○	○	○	◉	○	essential

선택해제

응답을 수정 중입니다. 이 페이지의 URL을 공유하면 다른 사용자도 내 응답을 수정할 수 있습니다.

새 응답 작성하기

Does your paper address subitem 1b-v?

Copy and paste relevant sections from the manuscript abstract (include quotes in quotation marks "like this" to indicate direct quotes from your manuscript), or elaborate on this item by providing additional information not in the ms, or briefly explain why the item is not applicable/relevant for your study

"In this small exploratory pilot study conducted under supervised laboratory conditions, the 6-week trajectories did not differ significantly between groups." The abstract also states that larger studies are required. Because direct use/adherence metrics were not available, the nonsignificant longitudinal result cannot be attributed to lack of intervention uptake.

INTRODUCTION

2a) In INTRODUCTION: Scientific background and explanation of rationale

2a-i) Problem and the type of system/solution

Describe the problem and the type of system/solution that is object of the study: intended as stand-alone intervention vs. incorporated in broader health care program? Intended for a particular patient population? Goals of the intervention, e.g., being more cost-effective to other interventions, replace or complement other solutions? (Note: Details about the intervention are provided in "Methods" under 5)

	1	2	3	4	5	
subitem not at all important	○	○	○	○	◉	essential

선택해제

응답을 수정 중입니다. 이 페이지의 URL을 공유하면 다른 사용자도 내 응답을 수정할 수 있습니다.

새 응답 작성하기

Does your paper address subitem 2a-i? *

Copy and paste relevant sections from the manuscript (include quotes in quotation marks "like this" to indicate direct quotes from your manuscript), or elaborate on this item by providing additional information not in the ms, or briefly explain why the item is not applicable/relevant for your study

"This exploratory study evaluated a webcam-based real-time visual feedback system during Baduanjin practice in older adults." The prototype used a standard RGB webcam and pose-tracking algorithm to calculate frontal-plane 2D projected joint-angle discrepancies relative to an expert guide video. The evaluated setting was supervised face-to-face laboratory use, not remote care.

2a-ii) Scientific background, rationale: What is known about the (type of) system

Scientific background, rationale: What is known about the (type of) system that is the object of the study (be sure to discuss the use of similar systems for other conditions/diagnoses, if appropiate), motivation for the study, i.e. what are the reasons for and what is the context for this specific study, from which stakeholder viewpoint is the study performed, potential impact of findings [2]. Briefly justify the choice of the comparator.

	1	2	3	4	5	
subitem not at all important	○	○	○	○	◉	essential

선택해제

Does your paper address subitem 2a-ii? *

Copy and paste relevant sections from the manuscript (include quotes in quotation marks "like this" to indicate direct quotes from your manuscript), or elaborate on this item by providing additional information not in the ms, or briefly explain why the item is not applicable/relevant for your study

The Introduction describes age-related proprioceptive decline and increased reliance on visual information, the potential benefits of Baduanjin, limitations of unaided self-directed movement, and the rationale for external visual feedback. It also discusses computer-vision–based posture assessment and attentional-focus/motor-learning literature. The comparator used the same guide video without supplementary real-time color-coded joint-discrepancy feedback.

응답을 수정 중입니다. 이 페이지의 URL을 공유하면 다른 사용자도 내 응답을 수정할 수 있습니다.

새 응답 작성하기

2b) In INTRODUCTION: Specific objectives or hypotheses

Does your paper address CONSORT subitem 2b? *

Copy and paste relevant sections from the manuscript (include quotes in quotation marks "like this" to indicate direct quotes from your manuscript), or elaborate on this item by providing additional information not in the ms, or briefly explain why the item is not applicable/relevant for your study

"To explore whether webcam-based real-time visual feedback delivered during supervised laboratory sessions was associated with differences in webcam-derived 2D pose discrepancy and movement consistency during Baduanjin practice in older adults."

METHODS

3a) Description of trial design (such as parallel, factorial) including allocation ratio

Does your paper address CONSORT subitem 3a? *

Copy and paste relevant sections from the manuscript (include quotes in quotation marks "like this" to indicate direct quotes from your manuscript), or elaborate on this item by providing additional information not in the ms, or briefly explain why the item is not applicable/relevant for your study

"This was a single-center, 2-arm, parallel-group exploratory pilot randomized study with a 1:1 allocation ratio.

3b) Important changes to methods after trial commencement (such as eligibility criteria), with reasons

응답을 수정 중입니다. 이 페이지의 URL을 공유하면 다른 사용자도 내 응답을 수정할 수 있습니다.

새 응답 작성하기

Does your paper address CONSORT subitem 3b? *

Copy and paste relevant sections from the manuscript (include quotes in quotation marks "like this" to indicate direct quotes from your manuscript), or elaborate on this item by providing additional information not in the ms, or briefly explain why the item is not applicable/relevant for your study

No important changes to eligibility criteria or trial methods after trial commencement are documented in the current manuscript. The statistical analysis was substantially revised after peer review, but this was a post hoc reanalysis of the existing data rather than a change to trial conduct.

3b-i) Bug fixes, Downtimes, Content Changes

Bug fixes, Downtimes, Content Changes: ehealth systems are often dynamic systems. A description of changes to methods therefore also includes important changes made on the intervention or comparator during the trial (e.g., major bug fixes or changes in the functionality or content) (5-iii) and other "unexpected events" that may have influenced study design such as staff changes, system failures/downtimes, etc. [2].

	1	2	3	4	5	
subitem not at all important	○	○	○	◉	○	essential

선택해제

Does your paper address subitem 3b-i?

Copy and paste relevant sections from the manuscript (include quotes in quotation marks "like this" to indicate direct quotes from your manuscript), or elaborate on this item by providing additional information not in the ms, or briefly explain why the item is not applicable/relevant for your study

The analytic workbook used for the current reanalysis did not contain systematic participant-level records of technical interruptions, protocol deviations, or system downtimes. Therefore, no unsupported statement about the presence or absence of such events is made.

4a) Eligibility criteria for participants

응답을 수정 중입니다. 이 페이지의 URL을 공유하면 다른 사용자도 내 응답을 수정할 수 있습니다.

새 응답 작성하기

Does your paper address CONSORT subitem 4a? *

Copy and paste relevant sections from the manuscript (include quotes in quotation marks "like this" to indicate direct quotes from your manuscript), or elaborate on this item by providing additional information not in the ms, or briefly explain why the item is not applicable/relevant for your study

"Eligibility criteria were adults aged 60 years or older; ability to stand independently and perform low-intensity Baduanjin movements without physical assistance; ability to understand verbal or visual instructions; sufficient visual and auditory ability to perceive the guide video and feedback interface; no regular Baduanjin or similar structured mind-body training during the preceding 3 months; willingness to complete the 6-week program; and capacity to provide written informed consent." The manuscript also lists the corresponding exclusion criteria.

4a-i) Computer / Internet literacy

Computer / Internet literacy is often an implicit "de facto" eligibility criterion - this should be explicitly clarified.

	1	2	3	4	5	
subitem not at all important	○	○	◉	○	○	essential

선택해제

Does your paper address subitem 4a-i?

Copy and paste relevant sections from the manuscript (include quotes in quotation marks "like this" to indicate direct quotes from your manuscript), or elaborate on this item by providing additional information not in the ms, or briefly explain why the item is not applicable/relevant for your study

Computer or Internet literacy was not an eligibility criterion because participants did not independently operate an online intervention. The system was operated by study staff during supervised laboratory sessions. Participants were required to understand verbal or visual instructions and to perceive the guide video and feedback interface

응답을 수정 중입니다. 이 페이지의 URL을 공유하면 다른 사용자도 내 응답을 수정할 수 있습니다.

새 응답 작성하기

4a-ii) Open vs. closed, web-based vs. face-to-face assessments:

Open vs. closed, web-based vs. face-to-face assessments: Mention how participants were recruited (online vs. offline), e.g., from an open access website or from a clinic, and clarify if this was a purely web-based trial, or there were face-to-face components (as part of the intervention or for assessment), i.e., to what degree got the study team to know the participant. In online-only trials, clarify if participants were quasi-anonymous and whether having multiple identities was possible or whether technical or logistical measures (e.g., cookies, email confirmation, phone calls) were used to detect/prevent these.

	1	2	3	4	5	
subitem not at all important	○	○	○	○	◉	essential

선택해제

Does your paper address subitem 4a-ii? *

Copy and paste relevant sections from the manuscript (include quotes in quotation marks "like this" to indicate direct quotes from your manuscript), or elaborate on this item by providing additional information not in the ms, or briefly explain why the item is not applicable/relevant for your study

All training and data collection were conducted face-to-face at the Konkuk University motion-analysis laboratory with study staff present. The intervention was not publicly accessible for online self-enrollment. The exact recruitment channel is not reported in the manuscript.

4a-iii) Information giving during recruitment

Information given during recruitment. Specify how participants were briefed for recruitment and in the informed consent procedures (e.g., publish the informed consent documentation as appendix, see also item X26), as this information may have an effect on user self-selection, user expectation and may also bias results.

	1	2	3	4	5	
subitem not at all important	○	○	○	◉	○	essential

선택해제

응답을 수정 중입니다. 이 페이지의 URL을 공유하면 다른 사용자도 내 응답을 수정할 수 있습니다.

새 응답 작성하기

Does your paper address subitem 4a-iii?

Copy and paste relevant sections from the manuscript (include quotes in quotation marks "like this" to indicate direct quotes from your manuscript), or elaborate on this item by providing additional information not in the ms, or briefly explain why the item is not applicable/relevant for your study

The manuscript states that written informed consent was obtained before participation. More detailed recruitment briefing materials or the exact information presented during recruitment are not reported in the current manuscript.

4b) Settings and locations where the data were collected

Does your paper address CONSORT subitem 4b? *

Copy and paste relevant sections from the manuscript (include quotes in quotation marks "like this" to indicate direct quotes from your manuscript), or elaborate on this item by providing additional information not in the ms, or briefly explain why the item is not applicable/relevant for your study

All training and data collection were conducted face-to-face at the Konkuk University motion-analysis laboratory from July to September 2025, with study staff operating the system and monitoring participants

4b-i) Report if outcomes were (self-)assessed through online questionnaires

Clearly report if outcomes were (self-)assessed through online questionnaires (as common in web-based trials) or otherwise.

	1	2	3	4	5	
subitem not at all important	○	○	◉	○	○	essential

선택해제

응답을 수정 중입니다. 이 페이지의 URL을 공유하면 다른 사용자도 내 응답을 수정할 수 있습니다.

새 응답 작성하기

Does your paper address subitem 4b-i? *

Copy and paste relevant sections from the manuscript (include quotes in quotation marks "like this" to indicate direct quotes from your manuscript), or elaborate on this item by providing additional information not in the ms, or briefly explain why the item is not applicable/relevant for your study

Not applicable. Outcomes were not self-assessed through online questionnaires. The principal outcome was webcam-derived 2D pose discrepancy calculated from recorded movement data.

4b-ii) Report how institutional affiliations are displayed

Report how institutional affiliations are displayed to potential participants [on ehealth media], as affiliations with prestigious hospitals or universities may affect volunteer rates, use, and reactions with regards to an intervention.(Not a required item – describe only if this may bias results)

	1	2	3	4	5	
subitem not at all important	○	◉	○	○	○	essential

선택해제

Does your paper address subitem 4b-ii?

Copy and paste relevant sections from the manuscript (include quotes in quotation marks "like this" to indicate direct quotes from your manuscript), or elaborate on this item by providing additional information not in the ms, or briefly explain why the item is not applicable/relevant for your study

Not applicable/not reported. This was not an open web-based recruitment or intervention platform, and the manuscript does not describe institutional branding displayed to potential participants through eHealth media.

5) The interventions for each group with sufficient details to allow replication, including how and when they were actually administered

응답을 수정 중입니다. 이 페이지의 URL을 공유하면 다른 사용자도 내 응답을 수정할 수 있습니다.

새 응답 작성하기

5-i) Mention names, credential, affiliations of the developers, sponsors, and owners

Mention names, credential, affiliations of the developers, sponsors, and owners [6] (if authors/evaluators are owners or developer of the software, this needs to be declared in a “Conflict of interest” section or mentioned elsewhere in the manuscript).

	1	2	3	4	5	
subitem not at all important	○	○	○	◉	○	essential

선택해제

Does your paper address subitem 5-i?

Copy and paste relevant sections from the manuscript (include quotes in quotation marks "like this" to indicate direct quotes from your manuscript), or elaborate on this item by providing additional information not in the ms, or briefly explain why the item is not applicable/relevant for your study

The manuscript describes the intervention as a research prototype implemented in Unity 3D and used by the study team. Funding was provided by the Korea Institute of Oriental Medicine and the Korea Health Technology R&D Project through the Korea Health Industry Development Institute, funded by the Ministry of Health & Welfare, Republic of Korea. The manuscript does not separately identify a commercial owner or formal software developer entity. The authors declare no conflicts of interest.

5-ii) Describe the history/development process

Describe the history/development process of the application and previous formative evaluations (e.g., focus groups, usability testing), as these will have an impact on adoption/use rates and help with interpreting results.

	1	2	3	4	5	
subitem not at all important	○	○	◉	○	○	essential

선택해제

응답을 수정 중입니다. 이 페이지의 URL을 공유하면 다른 사용자도 내 응답을 수정할 수 있습니다.

새 응답 작성하기

Does your paper address subitem 5-ii?

Copy and paste relevant sections from the manuscript (include quotes in quotation marks "like this" to indicate direct quotes from your manuscript), or elaborate on this item by providing additional information not in the ms, or briefly explain why the item is not applicable/relevant for your study

The manuscript describes the implemented research prototype and its technical workflow but does not report a separate prior formative development program, focus groups, or pretrial usability testing.

5-iii) Revisions and updating

Revisions and updating. Clearly mention the date and/or version number of the application/intervention (and comparator, if applicable) evaluated, or describe whether the intervention underwent major changes during the evaluation process, or whether the development and/or content was “frozen” during the trial. Describe dynamic components such as news feeds or changing content which may have an impact on the replicability of the intervention (for unexpected events see item 3b).

	1	2	3	4	5	
subitem not at all important	○	○	○	○	◉	essential

선택해제

Does your paper address subitem 5-iii?

Copy and paste relevant sections from the manuscript (include quotes in quotation marks "like this" to indicate direct quotes from your manuscript), or elaborate on this item by providing additional information not in the ms, or briefly explain why the item is not applicable/relevant for your study

No formal software version number is reported in the manuscript, and no major intervention-content changes during the trial are documented.

응답을 수정 중입니다. 이 페이지의 URL을 공유하면 다른 사용자도 내 응답을 수정할 수 있습니다.

새 응답 작성하기

5-iv) Quality assurance methods

Provide information on quality assurance methods to ensure accuracy and quality of information provided [1], if applicable.

	1	2	3	4	5	
subitem not at all important	○	○	○	○	◉	essential

선택해제

Does your paper address subitem 5-iv?

Copy and paste relevant sections from the manuscript (include quotes in quotation marks "like this" to indicate direct quotes from your manuscript), or elaborate on this item by providing additional information not in the ms, or briefly explain why the item is not applicable/relevant for your study

Before measurement, participant-camera positioning was individually adjusted using an angle-based procedure. Approximately 90° elbow flexion and 45° knee flexion were confirmed with a manual goniometer, and the participant-camera relationship was adjusted until webcam-derived angles matched these reference angles as closely as possible. Before each session, staff checked full-body and major-joint visibility. If camera position or full-body visibility was disturbed, recording was paused and the positioning check was repeated.

5-v) Ensure replicability by publishing the source code, and/or providing screenshots/screen-capture video, and/or providing flowcharts of the algorithms used

Ensure replicability by publishing the source code, and/or providing screenshots/screen-capture video, and/or providing flowcharts of the algorithms used. Replicability (i.e., other researchers should in principle be able to replicate the study) is a hallmark of scientific reporting.

	1	2	3	4	5	
subitem not at all important	○	○	○	○	◉	essential

선택해제

응답을 수정 중입니다. 이 페이지의 URL을 공유하면 다른 사용자도 내 응답을 수정할 수 있습니다.

새 응답 작성하기

Does your paper address subitem 5-v?

Copy and paste relevant sections from the manuscript (include quotes in quotation marks "like this" to indicate direct quotes from your manuscript), or elaborate on this item by providing additional information not in the ms, or briefly explain why the item is not applicable/relevant for your study

The manuscript provides a deidentified system screenshot (Figure 1), the MediaPipe-based landmark approach, the projected 3-point joint-angle equation, the camera setup, frame-by-frame comparison method, and data-quality handling. Source code is not published in the current manuscript

5-vi) Digital preservation

Digital preservation: Provide the URL of the application, but as the intervention is likely to change or disappear over the course of the years; also make sure the intervention is archived (Internet Archive, webcitation.org, and/or publishing the source code or screenshots/videos alongside the article). As pages behind login screens cannot be archived, consider creating demo pages which are accessible without login.

	1	2	3	4	5	
subitem not at all important	○	○	○	◉	○	essential

선택해제

Does your paper address subitem 5-vi?

Copy and paste relevant sections from the manuscript (include quotes in quotation marks "like this" to indicate direct quotes from your manuscript), or elaborate on this item by providing additional information not in the ms, or briefly explain why the item is not applicable/relevant for your study

No public intervention URL or archived software version is reported. A deidentified screenshot is preserved in Figure 1. Source code and a public demo/archive are not provided in the current manuscript.

응답을 수정 중입니다. 이 페이지의 URL을 공유하면 다른 사용자도 내 응답을 수정할 수 있습니다.

새 응답 작성하기

5-vii) Access

Access: Describe how participants accessed the application, in what setting/context, if they had to pay (or were paid) or not, whether they had to be a member of specific group. If known, describe how participants obtained “access to the platform and Internet” [1]. To ensure access for editors/reviewers/readers, consider to provide a “backdoor” login account or demo mode for reviewers/readers to explore the application (also important for archiving purposes, see vi).

	1	2	3	4	5	
subitem not at all important	○	○	○	○	◉	essential

선택해제

Does your paper address subitem 5-vii? *

Copy and paste relevant sections from the manuscript (include quotes in quotation marks "like this" to indicate direct quotes from your manuscript), or elaborate on this item by providing additional information not in the ms, or briefly explain why the item is not applicable/relevant for your study

"The prototype was operated on a local study workstation and was not publicly accessible." Participants accessed the intervention only during supervised laboratory sessions; study staff operated the system.

응답을 수정 중입니다. 이 페이지의 URL을 공유하면 다른 사용자도 내 응답을 수정할 수 있습니다.

새 응답 작성하기

5-viii) Mode of delivery, features/functionalities/components of the intervention and comparator, and the theoretical framework

Describe mode of delivery, features/functionalities/components of the intervention and comparator, and the theoretical framework [6] used to design them (instructional strategy [1], behaviour change techniques, persuasive features, etc., see e.g., [7, 8] for terminology). This includes an in-depth description of the content (including where it is coming from and who developed it) [1]," whether [and how] it is tailored to individual circumstances and allows users to track their progress and receive feedback" [6]. This also includes a description of communication delivery channels and – if computer-mediated communication is a component – whether communication was synchronous or asynchronous [6]. It also includes information on presentation strategies [1], including page design principles, average amount of text on pages, presence of hyperlinks to other resources, etc. [1].

	1	2	3	4	5	
subitem not at all important	○	○	○	○	◉	essential

선택해제

Does your paper address subitem 5-viii? *

Copy and paste relevant sections from the manuscript (include quotes in quotation marks "like this" to indicate direct quotes from your manuscript), or elaborate on this item by providing additional information not in the ms, or briefly explain why the item is not applicable/relevant for your study

The research prototype extracted participant landmarks from a frontal RGB webcam using MediaPipe Pose, calculated projected 2D joint-angle discrepancies relative to an expert guide video, and displayed real-time color-coded feedback. The feedback group viewed the Baduanjin guide video plus real-time color-coded joint-discrepancy feedback. The nonfeedback group viewed the same standard guide video without supplementary real-time feedback. The Introduction discusses external visual anchors and attentional-focus/motor-learning concepts, but attentional focus was not experimentally isolated as a mechanism.

응답을 수정 중입니다. 이 페이지의 URL을 공유하면 다른 사용자도 내 응답을 수정할 수 있습니다.

새 응답 작성하기

5-ix) Describe use parameters

Describe use parameters (e.g., intended “doses” and optimal timing for use). Clarify what instructions or recommendations were given to the user, e.g., regarding timing, frequency, heaviness of use, if any, or was the intervention used ad libitum.

	1	2	3	4	5	
subitem not at all important	○	○	○	○	◉	essential

선택해제

Does your paper address subitem 5-ix?

Copy and paste relevant sections from the manuscript (include quotes in quotation marks "like this" to indicate direct quotes from your manuscript), or elaborate on this item by providing additional information not in the ms, or briefly explain why the item is not applicable/relevant for your study

"Sessions were scheduled once weekly for 6 weeks, and the Baduanjin sequence lasted 409 seconds per performance."

5-x) Clarify the level of human involvement

Clarify the level of human involvement (care providers or health professionals, also technical assistance) in the e-intervention or as co-intervention (detail number and expertise of professionals involved, if any, as well as “type of assistance offered, the timing and frequency of the support, how it is initiated, and the medium by which the assistance is delivered”. It may be necessary to distinguish between the level of human involvement required for the trial, and the level of human involvement required for a routine application outside of a RCT setting (discuss under item 21 – generalizability).

	1	2	3	4	5	
subitem not at all important	○	○	○	○	◉	essential

선택해제

응답을 수정 중입니다. 이 페이지의 URL을 공유하면 다른 사용자도 내 응답을 수정할 수 있습니다. 새 응답 작성하기

Does your paper address subitem 5-x?

Copy and paste relevant sections from the manuscript (include quotes in quotation marks "like this" to indicate direct quotes from your manuscript), or elaborate on this item by providing additional information not in the ms, or briefly explain why the item is not applicable/relevant for your study

Study staff operated the system and monitored participant safety throughout all supervised laboratory sessions. Participants did not use the system remotely or without supervision.

5-xi) Report any prompts/reminders used

Report any prompts/reminders used: Clarify if there were prompts (letters, emails, phone calls, SMS) to use the application, what triggered them, frequency etc. It may be necessary to distinguish between the level of prompts/reminders required for the trial, and the level of prompts/reminders for a routine application outside of a RCT setting (discuss under item 21 – generalizability).

	1	2	3	4	5	
subitem not at all important	○	○	◉	○	○	essential

선택해제

Does your paper address subitem 5-xi? *

Copy and paste relevant sections from the manuscript (include quotes in quotation marks "like this" to indicate direct quotes from your manuscript), or elaborate on this item by providing additional information not in the ms, or briefly explain why the item is not applicable/relevant for your study

No scheduled email, SMS, telephone, or app-based prompts/reminders are reported in the manuscript.

응답을 수정 중입니다. 이 페이지의 URL을 공유하면 다른 사용자도 내 응답을 수정할 수 있습니다.

새 응답 작성하기

5-xii) Describe any co-interventions (incl. training/support)

Describe any co-interventions (incl. training/support): Clearly state any interventions that are provided in addition to the targeted eHealth intervention, as ehealth intervention may not be designed as stand-alone intervention. This includes training sessions and support [1]. It may be necessary to distinguish between the level of training required for the trial, and the level of training for a routine application outside of a RCT setting (discuss under item 21 – generalizability.

	1	2	3	4	5	
subitem not at all important	○	○	○	○	◉	essential

선택해제

Does your paper address subitem 5-xii? *

Copy and paste relevant sections from the manuscript (include quotes in quotation marks "like this" to indicate direct quotes from your manuscript), or elaborate on this item by providing additional information not in the ms, or briefly explain why the item is not applicable/relevant for your study

Both groups performed the same Baduanjin guide-video sequence during supervised laboratory sessions. The feedback group additionally received real-time color-coded joint-discrepancy feedback; the nonfeedback group did not receive this supplementary feedback. Study staff operated the system and monitored safety.

6a) Completely defined pre-specified primary and secondary outcome measures, including how and when they were assessed

응답을 수정 중입니다. 이 페이지의 URL을 공유하면 다른 사용자도 내 응답을 수정할 수 있습니다.

새 응답 작성하기

Does your paper address CONSORT subitem 6a? *

Copy and paste relevant sections from the manuscript (include quotes in quotation marks "like this" to indicate direct quotes from your manuscript), or elaborate on this item by providing additional information not in the ms, or briefly explain why the item is not applicable/relevant for your study

The study was exploratory and no confirmatory prespecified primary clinical outcome is documented. The measured kinematic outcome was the absolute discrepancy, in degrees, between participant and expert-guide joint angles projected in the frontal 2D image plane. Frame-level discrepancies were aggregated to participant-level session and weekly summaries. Joint- and movement-specific 6-week means and longitudinal variability/error-drift measures were analyzed exploratorily.

6a-i) Online questionnaires: describe if they were validated for online use and apply CHERRIES items to describe how the questionnaires were designed/deployed

If outcomes were obtained through online questionnaires, describe if they were validated for online use and apply CHERRIES items to describe how the questionnaires were designed/deployed [9].

	1	2	3	4	5	
subitem not at all important	○	○	◉	○	○	essential

선택해제

Does your paper address subitem 6a-i?

Copy and paste relevant sections from manuscript text

Not applicable. No study outcome was obtained through an online questionnaire.

응답을 수정 중입니다. 이 페이지의 URL을 공유하면 다른 사용자도 내 응답을 수정할 수 있습니다.

새 응답 작성하기

6a-ii) Describe whether and how “use” (including intensity of use/dosage) was defined/measured/monitored

Describe whether and how “use” (including intensity of use/dosage) was defined/measured/monitored (logins, logfile analysis, etc.). Use/adoption metrics are important process outcomes that should be reported in any ehealth trial.

	1	2	3	4	5	
subitem not at all important	○	○	○	○	◉	essential

선택해제

Does your paper address subitem 6a-ii?

Copy and paste relevant sections from manuscript text

The scheduled intervention exposure was once weekly for 6 weeks, with a 409-second Baduanjin performance per session. The analytic workbook did not contain systematic participant-level measures of session completion, intervention exposure, logins, or comparable usage metrics; these were therefore not quantitatively analyzed.

6a-iii) Describe whether, how, and when qualitative feedback from participants was obtained

Describe whether, how, and when qualitative feedback from participants was obtained (e.g., through emails, feedback forms, interviews, focus groups).

	1	2	3	4	5	
subitem not at all important	○	○	◉	○	○	essential

선택해제

Does your paper address subitem 6a-iii?

Copy and paste relevant sections from manuscript text

응답을 수정 중입니다. 이 페이지의 URL을 공유하면 다른 사용자도 내 응답을 수정할 수 있습니다.

새 응답 작성하기

6b) Any changes to trial outcomes after the trial commenced, with reasons

Does your paper address CONSORT subitem 6b? *

Copy and paste relevant sections from the manuscript (include quotes in quotation marks "like this" to indicate direct quotes from your manuscript), or elaborate on this item by providing additional information not in the ms, or briefly explain why the item is not applicable/relevant for your study

No changes to trial outcomes during trial conduct are documented. Following peer review, the existing data were reanalyzed using longitudinal mixed-effects models and multiplicity adjustment, and interpretation was revised accordingly; this did not change the data collected during the trial.

7a) How sample size was determined

NPT: When applicable, details of whether and how the clustering by care provides or centers was addressed

7a-i) Describe whether and how expected attrition was taken into account when calculating the sample size

Describe whether and how expected attrition was taken into account when calculating the sample size.

	1	2	3	4	5	
subitem not at all important	○	○	○	◉	○	essential

선택해제

응답을 수정 중입니다. 이 페이지의 URL을 공유하면 다른 사용자도 내 응답을 수정할 수 있습니다.

새 응답 작성하기

Does your paper address subitem 7a-i?

Copy and paste relevant sections from manuscript title (include quotes in quotation marks "like this" to indicate direct quotes from your manuscript), or elaborate on this item by providing additional information not in the ms, or briefly explain why the item is not applicable/relevant for your study

"No formal power-based sample-size calculation was performed. The sample was determined by the number of eligible participants who could be recruited and complete the study during the available period. The study was therefore exploratory and was not powered to establish definitive clinical effectiveness."

7b) When applicable, explanation of any interim analyses and stopping guidelines

Does your paper address CONSORT subitem 7b? *

Copy and paste relevant sections from the manuscript (include quotes in quotation marks "like this" to indicate direct quotes from your manuscript), or elaborate on this item by providing additional information not in the ms, or briefly explain why the item is not applicable/relevant for your study

No interim analyses or formal stopping guidelines are reported; the study was a small 6-week exploratory pilot study.

8a) Method used to generate the random allocation sequence

NPT: When applicable, how care providers were allocated to each trial group

Does your paper address CONSORT subitem 8a? *

Copy and paste relevant sections from the manuscript (include quotes in quotation marks "like this" to indicate direct quotes from your manuscript), or elaborate on this item by providing additional information not in the ms, or briefly explain why the item is not applicable/relevant for your study

"Participants were assigned in a 1:1 ratio using a computer-generated random sequence."

응답을 수정 중입니다. 이 페이지의 URL을 공유하면 다른 사용자도 내 응답을 수정할 수 있습니다.

새 응답 작성하기

8b) Type of randomisation; details of any restriction (such as blocking and block size)

Does your paper address CONSORT subitem 8b? *

Copy and paste relevant sections from the manuscript (include quotes in quotation marks "like this" to indicate direct quotes from your manuscript), or elaborate on this item by providing additional information not in the ms, or briefly explain why the item is not applicable/relevant for your study

A 1:1 random allocation is reported. Blocking, stratification, block size, or other restrictions are not described in the current manuscript.

9) Mechanism used to implement the random allocation sequence (such as sequentially numbered containers), describing any steps taken to conceal the sequence until interventions were assigned

Does your paper address CONSORT subitem 9? *

Copy and paste relevant sections from the manuscript (include quotes in quotation marks "like this" to indicate direct quotes from your manuscript), or elaborate on this item by providing additional information not in the ms, or briefly explain why the item is not applicable/relevant for your study

The current manuscript does not report the allocation-concealment mechanism

10) Who generated the random allocation sequence, who enrolled participants, and who assigned participants to interventions

응답을 수정 중입니다. 이 페이지의 URL을 공유하면 다른 사용자도 내 응답을 수정할 수 있습니다.

새 응답 작성하기

Does your paper address CONSORT subitem 10? *

Copy and paste relevant sections from the manuscript (include quotes in quotation marks "like this" to indicate direct quotes from your manuscript), or elaborate on this item by providing additional information not in the ms, or briefly explain why the item is not applicable/relevant for your study

The current manuscript records that participants were assigned in a 1:1 ratio using a computer-generated random sequence; however, the available study records reviewed for this revision do not specify who generated the allocation sequence, who enrolled participants, or who assigned participants to the intervention groups. These implementation roles should be confirmed from the original study records before final submission.

11a) If done, who was blinded after assignment to interventions (for example, participants, care providers, those assessing outcomes) and how

NPT: Whether or not administering co-interventions were blinded to group assignment

11a-i) Specify who was blinded, and who wasn't

Specify who was blinded, and who wasn't. Usually, in web-based trials it is not possible to blind the participants [1, 3] (this should be clearly acknowledged), but it may be possible to blind outcome assessors, those doing data analysis or those administering co-interventions (if any).

	1	2	3	4	5	
subitem not at all important	○	○	○	○	◉	essential

선택해제

Does your paper address subitem 11a-i? *

Copy and paste relevant sections from the manuscript (include quotes in quotation marks "like this" to indicate direct quotes from your manuscript), or elaborate on this item by providing additional information not in the ms, or briefly explain why the item is not applicable/relevant for your study

"Participants and session staff could not be blinded because the feedback interface was

응답을 수정 중입니다. 이 페이지의 URL을 공유하면 다른 사용자도 내 응답을 수정할 수 있습니다.

새 응답 작성하기

11a-ii) Discuss e.g., whether participants knew which intervention was the "intervention of interest" and which one was the "comparator"

Informed consent procedures (4a-ii) can create biases and certain expectations - discuss e.g., whether participants knew which intervention was the "intervention of interest" and which one was the "comparator".

	1	2	3	4	5	
subitem not at all important	○	○	○	◉	○	essential

선택해제

Does your paper address subitem 11a-ii?

Copy and paste relevant sections from the manuscript (include quotes in quotation marks "like this" to indicate direct quotes from your manuscript), or elaborate on this item by providing additional information not in the ms, or briefly explain why the item is not applicable/relevant for your study

Participants necessarily knew whether supplementary real-time color-coded feedback was visible during their session. The manuscript does not document whether participants were explicitly told which condition was considered the intervention of primary interest versus comparator.

11b) If relevant, description of the similarity of interventions

(this item is usually not relevant for ehealth trials as it refers to similarity of a placebo or sham intervention to a active medication/intervention)

Does your paper address CONSORT subitem 11b? *

Copy and paste relevant sections from the manuscript (include quotes in quotation marks "like this" to indicate direct quotes from your manuscript), or elaborate on this item by providing additional information not in the ms, or briefly explain why the item is not applicable/relevant for your study

Both groups viewed the same Baduanjin guide video and followed the same once-weekly 6-week schedule. The only intended group-specific component was that the feedback group

응답을 수정 중입니다. 이 페이지의 URL을 공유하면 다른 사용자도 내 응답을 수정할 수 있습니다.

새 응답 작성하기

12a) Statistical methods used to compare groups for primary and secondary outcomes

NPT: When applicable, details of whether and how the clustering by care providers or centers was addressed

Does your paper address CONSORT subitem 12a? *

Copy and paste relevant sections from the manuscript (include quotes in quotation marks "like this" to indicate direct quotes from your manuscript), or elaborate on this item by providing additional information not in the ms, or briefly explain why the item is not applicable/relevant for your study

Weekly mean 2D pose-discrepancy values were analyzed with a linear mixed-effects model including group, categorical week, and group-by-week interaction as fixed effects and a participant-specific random intercept. A Gaussian generalized estimating equation with exchangeable working correlation and robust covariance was used as a sensitivity analysis. Week-, joint-, and movement-specific comparisons were exploratory; Welch independent-samples t tests were used for joint- and movement-specific participant-level 6-week means. Holm correction was applied across 24 exploratory contrasts. Hedges g and 95% CIs were reported. Supportive repeated-joint/repeated-movement mixed models and participant-level error-drift analyses were also performed.

12a-i) Imputation techniques to deal with attrition / missing values

Imputation techniques to deal with attrition / missing values: Not all participants will use the intervention/comparator as intended and attrition is typically high in ehealth trials. Specify how participants who did not use the application or dropped out from the trial were treated in the statistical analysis (a complete case analysis is strongly discouraged, and simple imputation techniques such as LOCF may also be problematic [4]).

	1	2	3	4	5	
subitem not at all important	○	○	○	○	◉	essential

선택해제

응답을 수정 중입니다. 이 페이지의 URL을 공유하면 다른 사용자도 내 응답을 수정할 수 있습니다.

새 응답 작성하기

Does your paper address subitem 12a-i? *

Copy and paste relevant sections from the manuscript (include quotes in quotation marks "like this" to indicate direct quotes from your manuscript), or elaborate on this item by providing additional information not in the ms, or briefly explain why the item is not applicable/relevant for your study

"The final analytic matrices contained no missing outcome values among the 28 analyzed participants (168 weekly, 224 joint, and 280 movement observations were complete). Therefore, missing-data imputation was not required within this complete-case dataset." However, the 3 excluded participants were absent from the analytic workbook; therefore, their missingness mechanism, multiple imputation, intention-to-treat analysis, and attrition sensitivity could not be evaluated.

12b) Methods for additional analyses, such as subgroup analyses and adjusted analyses

Does your paper address CONSORT subitem 12b? *

Copy and paste relevant sections from the manuscript (include quotes in quotation marks "like this" to indicate direct quotes from your manuscript), or elaborate on this item by providing additional information not in the ms, or briefly explain why the item is not applicable/relevant for your study

Additional exploratory analyses included week-specific contrasts, joint-specific and movement-specific comparisons with global Holm correction, supportive repeated-joint and repeated-movement mixed models, participant-specific weekly slopes, within-participant SD/range analyses, and a random-intercept/random-slope model. A valid age-adjusted model could not be fitted because participant-level age was unavailable in the analytic workbook.

X26) REB/IRB Approval and Ethical Considerations [recommended as subheading under "Methods"] (not a CONSORT item)

응답을 수정 중입니다. 이 페이지의 URL을 공유하면 다른 사용자도 내 응답을 수정할 수 있습니다.

새 응답 작성하기

X26-i) Comment on ethics committee approval

	1	2	3	4	5	
subitem not at all important	○	○	○	○	◉	essential

선택해제

Does your paper address subitem X26-i?

Copy and paste relevant sections from the manuscript (include quotes in quotation marks "like this" to indicate direct quotes from your manuscript), or elaborate on this item by providing additional information not in the ms, or briefly explain why the item is not applicable/relevant for your study

"Ethics approval was obtained from the Institutional Review Board of Konkuk University (KKUIRB-202507-HR-092; approved July 25, 2025)."

x26-ii) Outline informed consent procedures

Outline informed consent procedures e.g., if consent was obtained offline or online (how? Checkbox, etc.?), and what information was provided (see 4a-ii). See [6] for some items to be included in informed consent documents.

	1	2	3	4	5	
subitem not at all important	○	○	○	○	◉	essential

선택해제

Does your paper address subitem X26-ii?

Copy and paste relevant sections from the manuscript (include quotes in quotation marks "like this" to indicate direct quotes from your manuscript), or elaborate on this item by providing additional information not in the ms, or briefly explain why the item is not applicable/relevant for your study

"All participants provided written informed consent before participation." The current

응답을 수정 중입니다. 이 페이지의 URL을 공유하면 다른 사용자도 내 응답을 수정할 수 있습니다.

새 응답 작성하기

X26-iii) Safety and security procedures

Safety and security procedures, incl. privacy considerations, and any steps taken to reduce the likelihood or detection of harm (e.g., education and training, availability of a hotline)

	1	2	3	4	5	
subitem not at all important	○	○	○	○	◉	essential

선택해제

Does your paper address subitem X26-iii?

Copy and paste relevant sections from the manuscript (include quotes in quotation marks "like this" to indicate direct quotes from your manuscript), or elaborate on this item by providing additional information not in the ms, or briefly explain why the item is not applicable/relevant for your study

The prototype was operated on a local study workstation and was not publicly accessible. Study staff operated the system and monitored participant safety during sessions. Figure 1 was deidentified by obscuring facial features. Systematic participant-level adverse-event and technical-event metrics were not available in the analytic workbook and therefore were not quantitatively analyzed.

RESULTS

13a) For each group, the numbers of participants who were randomly assigned, received intended treatment, and were analysed for the primary outcome

NPT: The number of care providers or centers performing the intervention in each group and the number of patients treated by each care provider in each center

응답을 수정 중입니다. 이 페이지의 URL을 공유하면 다른 사용자도 내 응답을 수정할 수 있습니다.

새 응답 작성하기

Does your paper address CONSORT subitem 13a? *

Copy and paste relevant sections from the manuscript (include quotes in quotation marks "like this" to indicate direct quotes from your manuscript), or elaborate on this item by providing additional information not in the ms, or briefly explain why the item is not applicable/relevant for your study

Thirty-one participants were enrolled. The current complete-case analytic dataset contained 28 participants: feedback group n=14 and nonfeedback group n=14. The current records do not establish the timing of the 1 withdrawal and 2 incomplete records relative to randomization/allocation; therefore, the exact number randomized before these records became unavailable cannot be reconstructed without source records.

13b) For each group, losses and exclusions after randomisation, together with reasons

Does your paper address CONSORT subitem 13b? (NOTE: Preferably, this is shown in a CONSORT flow diagram) *

Copy and paste relevant sections from the manuscript (include quotes in quotation marks "like this" to indicate direct quotes from your manuscript), or elaborate on this item by providing additional information not in the ms, or briefly explain why the item is not applicable/relevant for your study

"One withdrawal and 2 incomplete records were documented. However, the timing of these 3 records relative to randomization/allocation and their group-specific assignment were not available in the current analytic dataset." Figure 2 therefore presents these records as a separate analytic-data note rather than assigning them to an unsupported pre- or post-randomization stage.

13b-i) Attrition diagram

Strongly recommended: An attrition diagram (e.g., proportion of participants still logging in or using the intervention/comparator in each group plotted over time, similar to a survival curve) or other figures or tables demonstrating usage/dose/engagement.

	1	2	3	4	5	
subitem not at all important	○	○	○	●	○	essential

응답을 수정 중입니다. 이 페이지의 URL을 공유하면 다른 사용자도 내 응답을 수정할 수 있습니다.

새 응답 작성하기

Does your paper address subitem 13b-i?

Copy and paste relevant sections from the manuscript or cite the figure number if applicable (include quotes in quotation marks "like this" to indicate direct quotes from your manuscript), or elaborate on this item by providing additional information not in the ms, or briefly explain why the item is not applicable/relevant for your study

Figure 2 provides the participant analytic-flow diagram. A longitudinal usage/engagement attrition diagram was not produced because systematic session-by-session usage/adherence data were not available in the analytic dataset.

14a) Dates defining the periods of recruitment and follow-up

Does your paper address CONSORT subitem 14a? *

Copy and paste relevant sections from the manuscript (include quotes in quotation marks "like this" to indicate direct quotes from your manuscript), or elaborate on this item by providing additional information not in the ms, or briefly explain why the item is not applicable/relevant for your study

"All training and data collection were conducted face-to-face at the Konkuk University motion-analysis laboratory from July to September 2025."

14a-i) Indicate if critical “secular events” fell into the study period

Indicate if critical “secular events” fell into the study period, e.g., significant changes in Internet resources available or “changes in computer hardware or Internet delivery resources”

	1	2	3	4	5	
subitem not at all important	○	○	◉	○	○	essential

선택해제

응답을 수정 중입니다. 이 페이지의 URL을 공유하면 다른 사용자도 내 응답을 수정할 수 있습니다.

새 응답 작성하기

Does your paper address subitem 14a-i?

Copy and paste relevant sections from the manuscript (include quotes in quotation marks "like this" to indicate direct quotes from your manuscript), or elaborate on this item by providing additional information not in the ms, or briefly explain why the item is not applicable/relevant for your study

No critical secular event or major change in hardware/Internet-delivery resources during the study period is documented in the manuscript.

14b) Why the trial ended or was stopped (early)

Does your paper address CONSORT subitem 14b? *

Copy and paste relevant sections from the manuscript (include quotes in quotation marks "like this" to indicate direct quotes from your manuscript), or elaborate on this item by providing additional information not in the ms, or briefly explain why the item is not applicable/relevant for your study

The manuscript does not report early stopping. The study was conducted as a planned 6-week exploratory pilot study.

15) A table showing baseline demographic and clinical characteristics for each group

NPT: When applicable, a description of care providers (case volume, qualification, expertise, etc.) and centers (volume) in each group

Does your paper address CONSORT subitem 15? *

Copy and paste relevant sections from the manuscript (include quotes in quotation marks "like this" to indicate direct quotes from your manuscript), or elaborate on this item by providing additional information not in the ms, or briefly explain why the item is not applicable/relevant for your study

Table 2 reports baseline characteristics by group: gender, age, height, and weight. Baseline characteristics are presented descriptively and significance tests were not performed.

응답을 수정 중입니다. 이 페이지의 URL을 공유하면 다른 사용자도 내 응답을 수정할 수 있습니다.

새 응답 작성하기

15-i) Report demographics associated with digital divide issues

In ehealth trials it is particularly important to report demographics associated with digital divide issues, such as age, education, gender, social-economic status, computer/Internet/ehealth literacy of the participants, if known.

	1	2	3	4	5	
subitem not at all important	○	○	○	◉	○	essential

선택해제

Does your paper address subitem 15-i? *

Copy and paste relevant sections from the manuscript (include quotes in quotation marks "like this" to indicate direct quotes from your manuscript), or elaborate on this item by providing additional information not in the ms, or briefly explain why the item is not applicable/relevant for your study

Age and gender are reported. Education, socioeconomic status, computer/Internet literacy, and eHealth literacy were not reported. Computer/Internet literacy was not a required eligibility criterion because study staff operated the local prototype during supervised sessions.

16) For each group, number of participants (denominator) included in each analysis and whether the analysis was by original assigned groups

16-i) Report multiple "denominators" and provide definitions

Report multiple "denominators" and provide definitions: Report N's (and effect sizes) "across a range of study participation [and use] thresholds" [1], e.g., N exposed, N consented, N used more than x times, N used more than y weeks, N participants "used" the intervention/comparator at specific pre-defined time points of interest (in absolute and relative numbers per group). Always clearly define "use" of the intervention.

	1	2	3	4	5	
subitem not at all important	○	○	○	○	◉	essential

응답을 수정 중입니다. 이 페이지의 URL을 공유하면 다른 사용자도 내 응답을 수정할 수 있습니다.

새 응답 작성하기

Does your paper address subitem 16-i? *

Copy and paste relevant sections from the manuscript (include quotes in quotation marks "like this" to indicate direct quotes from your manuscript), or elaborate on this item by providing additional information not in the ms, or briefly explain why the item is not applicable/relevant for your study

Enrolled: n=31. Complete-case analytic dataset: n=28 (feedback n=14; nonfeedback n=14), corresponding to 90.3% analyzable-record retention. Among these 28 participants, the analytic matrices contained 168 weekly, 224 joint, and 280 movement observations. Separate denominators based on session-use or exposure thresholds could not be reported because systematic participant-level usage metrics were unavailable.

16-ii) Primary analysis should be intent-to-treat

Primary analysis should be intent-to-treat, secondary analyses could include comparing only "users", with the appropriate caveats that this is no longer a randomized sample (see 18-i).

	1	2	3	4	5	
subitem not at all important	○	○	○	○	●	essential

선택해제

Does your paper address subitem 16-ii?

Copy and paste relevant sections from the manuscript (include quotes in quotation marks "like this" to indicate direct quotes from your manuscript), or elaborate on this item by providing additional information not in the ms, or briefly explain why the item is not applicable/relevant for your study

The primary reanalysis was not an intention-to-treat analysis; it was a complete-case analysis of the 28 participants represented in the available analytic workbook. The 3 excluded participants were absent from the workbook, so a valid post hoc ITT analysis, multiple imputation, or attrition sensitivity analysis could not be reconstructed.

17a) For each primary and secondary outcome, results for each group, and the

응답을 수정 중입니다. 이 페이지의 URL을 공유하면 다른 사용자도 내 응답을 수정할 수 있습니다.

새 응답 작성하기

Does your paper address CONSORT subitem 17a? *

Copy and paste relevant sections from the manuscript (include quotes in quotation marks "like this" to indicate direct quotes from your manuscript), or elaborate on this item by providing additional information not in the ms, or briefly explain why the item is not applicable/relevant for your study

The longitudinal mixed-effects model showed no significant group-by-week interaction (Wald $\chi^2(5)=1.09$, P=.955) and no significant overall week effect (Wald $\chi^2(5)=6.40$, P=.270). Averaged across 6 weeks, the estimated marginal feedback-minus-nonfeedback difference was −1.20° (95% CI −2.38° to −0.02°; P=.046), but this marginal pilot finding was sensitive to analytic approach. No week-specific or joint-specific comparison remained significant after global Holm correction. Form 3 remained significant after correction (mean difference −3.70°, 95% CI −5.86° to −1.54°; Hedges g=−1.30; Holm-adjusted P=.044).

17a-i) Presentation of process outcomes such as metrics of use and intensity of use

In addition to primary/secondary (clinical) outcomes, the presentation of process outcomes such as metrics of use and intensity of use (dose, exposure) and their operational definitions is critical. This does not only refer to metrics of attrition (13-b) (often a binary variable), but also to more continuous exposure metrics such as “average session length”. These must be accompanied by a technical description how a metric like a “session” is defined (e.g., timeout after idle time) [1] (report under item 6a).

	1	2	3	4	5	
subitem not at all important	○	○	○	○	○	essential

Does your paper address subitem 17a-i?

Copy and paste relevant sections from the manuscript (include quotes in quotation marks "like this" to indicate direct quotes from your manuscript), or elaborate on this item by providing additional information not in the ms, or briefly explain why the item is not applicable/relevant for your study

Analyzable-record retention was 90.3% (28/31). Session completion, exposure, usability, acceptability, technical interruptions, protocol deviations, and adverse-event metrics were not systematically available in the analytic workbook and therefore were not quantitatively reported.

응답을 수정 중입니다. 이 페이지의 URL을 공유하면 다른 사용자도 내 응답을 수정할 수 있습니다.

새 응답 작성하기

17b) For binary outcomes, presentation of both absolute and relative effect sizes is recommended

Does your paper address CONSORT subitem 17b? *

Copy and paste relevant sections from the manuscript (include quotes in quotation marks "like this" to indicate direct quotes from your manuscript), or elaborate on this item by providing additional information not in the ms, or briefly explain why the item is not applicable/relevant for your study

Not applicable. The principal and exploratory outcomes reported in this study were continuous 2D pose-discrepancy and longitudinal variability measures rather than binary outcomes.

18) Results of any other analyses performed, including subgroup analyses and adjusted analyses, distinguishing pre-specified from exploratory

Does your paper address CONSORT subitem 18? *

Copy and paste relevant sections from the manuscript (include quotes in quotation marks "like this" to indicate direct quotes from your manuscript), or elaborate on this item by providing additional information not in the ms, or briefly explain why the item is not applicable/relevant for your study

Exploratory analyses included 6 week-specific contrasts, 8 joint-specific contrasts, and 10 movement-specific contrasts with global Holm familywise-error correction. Supportive repeated-joint and repeated-movement mixed models were fitted. Longitudinal error drift was directly explored using participant-specific slopes, within-participant SD/range, and a random-intercept/random-slope model. These analyses are explicitly identified as exploratory/sensitivity analyses.

응답을 수정 중입니다. 이 페이지의 URL을 공유하면 다른 사용자도 내 응답을 수정할 수 있습니다.

새 응답 작성하기

18-i) Subgroup analysis of comparing only users

A subgroup analysis of comparing only users is not uncommon in ehealth trials, but if done, it must be stressed that this is a self-selected sample and no longer an unbiased sample from a randomized trial (see 16-iii).

	1	2	3	4	5	
subitem not at all important	○	○	○	◉	○	essential

선택해제

Does your paper address subitem 18-i?

Copy and paste relevant sections from the manuscript (include quotes in quotation marks "like this" to indicate direct quotes from your manuscript), or elaborate on this item by providing additional information not in the ms, or briefly explain why the item is not applicable/relevant for your study

Not applicable. No subgroup analysis restricted to "users" or high-use participants was performed because systematic intervention-use thresholds were not available.

19) All important harms or unintended effects in each group
(for specific guidance see CONSORT for harms)

Does your paper address CONSORT subitem 19? *

Copy and paste relevant sections from the manuscript (include quotes in quotation marks "like this" to indicate direct quotes from your manuscript), or elaborate on this item by providing additional information not in the ms, or briefly explain why the item is not applicable/relevant for your study

Study staff monitored participant safety during all supervised sessions. However, systematic participant-level adverse-event data were not present in the analytic workbook; therefore, the manuscript does not make an unsupported claim that no adverse events occurred.

응답을 수정 중입니다. 이 페이지의 URL을 공유하면 다른 사용자도 내 응답을 수정할 수 있습니다.

새 응답 작성하기

19-i) Include privacy breaches, technical problems

Include privacy breaches, technical problems. This does not only include physical “harm” to participants, but also incidents such as perceived or real privacy breaches [1], technical problems, and other unexpected/unintended incidents. “Unintended effects” also includes unintended positive effects [2].

	1	2	3	4	5	
subitem not at all important	○	○	○	○	◉	essential

선택해제

Does your paper address subitem 19-i?

Copy and paste relevant sections from the manuscript (include quotes in quotation marks "like this" to indicate direct quotes from your manuscript), or elaborate on this item by providing additional information not in the ms, or briefly explain why the item is not applicable/relevant for your study

The prototype was local and not publicly accessible. Systematic participant-level records of privacy breaches, technical interruptions, or other technical incidents were not available in the analytic workbook and therefore were not quantitatively analyzed.

19-ii) Include qualitative feedback from participants or observations from staff/researchers

Include qualitative feedback from participants or observations from staff/researchers, if available, on strengths and shortcomings of the application, especially if they point to unintended/unexpected effects or uses. This includes (if available) reasons for why people did or did not use the application as intended by the developers.

	1	2	3	4	5	
subitem not at all important	○	○	◉	○	○	essential

선택해제

응답을 수정 중입니다. 이 페이지의 URL을 공유하면 다른 사용자도 내 응답을 수정할 수 있습니다.

새 응답 작성하기

Does your paper address subitem 19-ii?

Copy and paste relevant sections from the manuscript (include quotes in quotation marks "like this" to indicate direct quotes from your manuscript), or elaborate on this item by providing additional information not in the ms, or briefly explain why the item is not applicable/relevant for your study

No systematic qualitative participant feedback or staff-observation dataset is reported in the current manuscript.

DISCUSSION

22) Interpretation consistent with results, balancing benefits and harms, and considering other relevant evidence

NPT: In addition, take into account the choice of the comparator, lack of or partial blinding, and unequal expertise of care providers or centers in each group

22-i) Restate study questions and summarize the answers suggested by the data, starting with primary outcomes and process outcomes (use)

Restate study questions and summarize the answers suggested by the data, starting with primary outcomes and process outcomes (use).

	1	2	3	4	5	
subitem not at all important	○	○	○	○	◉	essential

선택해제

응답을 수정 중입니다. 이 페이지의 URL을 공유하면 다른 사용자도 내 응답을 수정할 수 있습니다.

새 응답 작성하기

Does your paper address subitem 22-i? *

Copy and paste relevant sections from the manuscript (include quotes in quotation marks "like this" to indicate direct quotes from your manuscript), or elaborate on this item by providing additional information not in the ms, or briefly explain why the item is not applicable/relevant for your study

The Discussion states that this exploratory pilot randomized study evaluated webcam-based real-time visual feedback during supervised Baduanjin practice in older adults. The group-by-week interaction was not significant, the average between-group difference was marginal and analysis-sensitive, no week-specific or joint-specific contrast survived global Holm correction, and Form 3 was the only multiplicity-adjusted exploratory signal. Direct analyses did not support prevention of longitudinal error drift.

22-ii) Highlight unanswered new questions, suggest future research

Highlight unanswered new questions, suggest future research.

	1	2	3	4	5	
subitem not at all important	○	○	○	◉	○	essential

선택해제

Does your paper address subitem 22-ii?

Copy and paste relevant sections from the manuscript (include quotes in quotation marks "like this" to indicate direct quotes from your manuscript), or elaborate on this item by providing additional information not in the ms, or briefly explain why the item is not applicable/relevant for your study

The manuscript recommends validating the 2D discrepancy measure against 3D motion capture or another criterion method, establishing test-retest reliability, evaluating usability, acceptability, technical reliability, adherence, and safety in real home settings, and conducting larger randomized studies to investigate attentional-focus or sensory-reweighting mechanisms.

응답을 수정 중입니다. 이 페이지의 URL을 공유하면 다른 사용자도 내 응답을 수정할 수 있습니다.

새 응답 작성하기

20-i) Typical limitations in ehealth trials

Typical limitations in ehealth trials: Participants in ehealth trials are rarely blinded. Ehealth trials often look at a multiplicity of outcomes, increasing risk for a Type I error. Discuss biases due to non-use of the intervention/usability issues, biases through informed consent procedures, unexpected events.

	1	2	3	4	5	
subitem not at all important	○	○	○	○	◉	essential

선택해제

Does your paper address subitem 20-i? *

Copy and paste relevant sections from the manuscript (include quotes in quotation marks "like this" to indicate direct quotes from your manuscript), or elaborate on this item by providing additional information not in the ms, or briefly explain why the item is not applicable/relevant for your study

The Limitations section addresses the small sample, lack of a confirmatory prespecified primary outcome/progression criterion/formal power calculation, lack of prospective registration, inability to perform ITT/missing-data sensitivity for the 3 unavailable participants, inability to fit an age-adjusted model, 2D single-camera measurement limitations and lack of criterion validity/test-retest reliability, multiplicity of 24 exploratory contrasts, nonsignificant direct error-drift analyses, and insufficiently characterized attendance/exposure/usability/technical-failure metrics.

21) Generalisability (external validity, applicability) of the trial findings

NPT: External validity of the trial findings according to the intervention, comparators, patients, and care providers or centers involved in the trial

응답을 수정 중입니다. 이 페이지의 URL을 공유하면 다른 사용자도 내 응답을 수정할 수 있습니다.

새 응답 작성하기

21-i) Generalizability to other populations

Generalizability to other populations: In particular, discuss generalizability to a general Internet population, outside of a RCT setting, and general patient population, including applicability of the study results for other organizations

	1	2	3	4	5	
subitem not at all important	○	○	○	○	◉	essential

선택해제

Does your paper address subitem 21-i?

Copy and paste relevant sections from the manuscript (include quotes in quotation marks "like this" to indicate direct quotes from your manuscript), or elaborate on this item by providing additional information not in the ms, or briefly explain why the item is not applicable/relevant for your study

The study was conducted in a supervised motion-analysis laboratory with staff operating the system. The manuscript explicitly states that it does not demonstrate unsupervised home feasibility or telerehabilitation effectiveness. Generalization to home use, remote care, other populations, or routine clinical implementation therefore requires separate validation.

21-ii) Discuss if there were elements in the RCT that would be different in a routine application setting

Discuss if there were elements in the RCT that would be different in a routine application setting (e.g., prompts/reminders, more human involvement, training sessions or other co-interventions) and what impact the omission of these elements could have on use, adoption, or outcomes if the intervention is applied outside of a RCT setting.

	1	2	3	4	5	
subitem not at all important	○	○	○	○	◉	essential

선택해제

응답을 수정 중입니다. 이 페이지의 URL을 공유하면 다른 사용자도 내 응답을 수정할 수 있습니다.

새 응답 작성하기

Does your paper address subitem 21-ii?

Copy and paste relevant sections from the manuscript (include quotes in quotation marks "like this" to indicate direct quotes from your manuscript), or elaborate on this item by providing additional information not in the ms, or briefly explain why the item is not applicable/relevant for your study

The evaluated protocol included face-to-face staff supervision, staff operation of the system, participant-specific camera positioning using goniometer-confirmed reference angles, pre-session visibility checks, and monitoring of participant safety. These elements may not be available in an unsupervised home setting; therefore, usability, technical reliability, safety, adherence, and effectiveness require separate real-world evaluation before routine home/telerehabilitation use is inferred.

OTHER INFORMATION

23) Registration number and name of trial registry

Does your paper address CONSORT subitem 23? *

Copy and paste relevant sections from the manuscript (include quotes in quotation marks "like this" to indicate direct quotes from your manuscript), or elaborate on this item by providing additional information not in the ms, or briefly explain why the item is not applicable/relevant for your study

"Trial Registration: Not registered." The Methods explains that the study was initially conceived as an exploratory pilot investigation focused on technical feasibility and preliminary kinematic evaluation rather than as a confirmatory efficacy trial; consequently, prospective trial registration was not undertaken before participant enrollment. The lack of prospective registration is acknowledged as a limitation.

24) Where the full trial protocol can be accessed, if available

응답을 수정 중입니다. 이 페이지의 URL을 공유하면 다른 사용자도 내 응답을 수정할 수 있습니다.

새 응답 작성하기

Does your paper address CONSORT subitem 24? *

Cite a Multimedia Appendix, other reference, or copy and paste relevant sections from the manuscript (include quotes in quotation marks "like this" to indicate direct quotes from your manuscript), or elaborate on this item by providing additional information not in the ms, or briefly explain why the item is not applicable/relevant for your study

"Trial Registration: Not registered." The Methods explains that the study was initially conceived as an exploratory pilot investigation focused on technical feasibility and preliminary kinematic evaluation rather than as a confirmatory efficacy trial; consequently, prospective trial registration was not undertaken before participant enrollment. The lack of prospective registration is acknowledged as a limitation.

25) Sources of funding and other support (such as supply of drugs), role of funders

Does your paper address CONSORT subitem 25? *

Copy and paste relevant sections from the manuscript (include quotes in quotation marks "like this" to indicate direct quotes from your manuscript), or elaborate on this item by providing additional information not in the ms, or briefly explain why the item is not applicable/relevant for your study

"This work was supported by the Korea Institute of Oriental Medicine (KSN2324022) and the Korea Health Technology R&D Project through the Korea Health Industry Development Institute, funded by the Ministry of Health & Welfare, Republic of Korea (RS-2023-KH138802). The funders had no role in study design, data collection, analysis, interpretation, manuscript preparation, or the decision to submit the manuscript."

X27) Conflicts of Interest (not a CONSORT item)

응답을 수정 중입니다. 이 페이지의 URL을 공유하면 다른 사용자도 내 응답을 수정할 수 있습니다.

새 응답 작성하기

X27-i) State the relation of the study team towards the system being evaluated

In addition to the usual declaration of interests (financial or otherwise), also state the relation of the study team towards the system being evaluated, i.e., state if the authors/evaluators are distinct from or identical with the developers/sponsors of the intervention.

	1	2	3	4	5	
subitem not at all important	○	○	○	○	◉	essential

선택해제

Does your paper address subitem X27-i?

Copy and paste relevant sections from the manuscript (include quotes in quotation marks "like this" to indicate direct quotes from your manuscript), or elaborate on this item by providing additional information not in the ms, or briefly explain why the item is not applicable/relevant for your study

The manuscript describes the intervention as a research prototype and states: "The authors declare no conflicts of interest." It does not separately identify a commercial owner or specify distinct developer-versus-evaluator roles.

About the CONSORT EHEALTH checklist

As a result of using this checklist, did you make changes in your manuscript? *

- ◉ yes, major changes
- ○ yes, minor changes
- ○ no

응답을 수정 중입니다. 이 페이지의 URL을 공유하면 다른 사용자도 내 응답을 수정할 수 있습니다.

새 응답 작성하기

What were the most important changes you made as a result of using this checklist?

The manuscript was substantially revised to distinguish supervised contact-free webcam measurement from telerehabilitation, describe the outcome as webcam-derived 2D pose discrepancy rather than validated anatomical kinematic accuracy, expand camera/setup and temporal-processing methods, clarify pilot/feasibility reporting and participant flow, replace separate week-by-week testing with participant-level longitudinal mixed-effects analysis, apply global Holm correction across 24 exploratory contrasts, add effect estimates and sensitivity analyses, directly test longitudinal error drift, moderate efficacy claims, expand limitations/generalizability, and add explicit trial-registration and generative-AI disclosures.

How much time did you spend on going through the checklist INCLUDING making changes in your manuscript *

I spent Approximately 4 hours

As a result of using this checklist, do you think your manuscript has improved? *

- (●) yes
- ( ) no
- ( ) 기타:

Would you like to become involved in the CONSORT EHEALTH group?

This would involve for example becoming involved in participating in a workshop and writing an "Explanation and Elaboration" document

- ( ) yes
- (●) no
- ( ) 기타:

응답을 수정 중입니다. 이 페이지의 URL을 공유하면 다른 사용자도 내 응답을 수정할 수 있습니다.

새 응답 작성하기

Any other comments or questions on CONSORT EHEALTH

The checklist was useful for identifying reporting gaps related to intervention access, human involvement, participant flow, process/use outcomes, reproducibility, and external validity. Several historical details could not be reconstructed from the available analytic dataset and are therefore reported transparently rather than inferred.

STOP - Save this form as PDF before you click submit

To generate a record that you filled in this form, we recommend to generate a PDF of this page (on a Mac, simply select "print" and then select "print as PDF") before you submit it.

When you submit your (revised) paper to JMIR, please upload the PDF as supplementary file.

Don't worry if some text in the textboxes is cut off, as we still have the complete information in our database. Thank you!

Final step: Click submit !

Click submit so we have your answers in our database!

제출

Google Forms를 통해 비밀번호를 제출하지 마세요.

이 콘텐츠는 Google이 만들거나 승인하지 않았습니다. - 양식 소유자에게 문의 - 서비스 약관 - 개인정보처리방침

양식이 의심스러운가요? 보고서

Google 설문지

응답을 수정 중입니다. 이 페이지의 URL을 공유하면 다른 사용자도 내 응답을 수정할 수 있습니다.

새 응답 작성하기

응답을 수정 중입니다. 이 페이지의 URL을 공유하면 다른 사용자도 내 응답을 수정할 수 있습니다.

새 응답 작성하기
